# The Interplay of Light and Microbial Symbiosis in Shaping Plant Economic Spectrum Strategies

**DOI:** 10.1007/s00248-026-02777-4

**Published:** 2026-05-08

**Authors:** Irene Ariadna De Lara-Del Rey, María Pérez-Fernández, Anathi Magadlela

**Affiliations:** 1 Department of Physical, Chemical and Natural Systems, Universidad Pablo de Olavide, Seville, 41701 Spain; 2 Centre for Global Change, Faculty of Natural and Applied Sciences, Sol Plaatje University, Private Bag X5008, Kimberley, 8300 South Africa; 3 School of Life Sciences, College of Agriculture, Engineering and Science, University of KwaZulu-Natal (Westville Campus), Private Bag X54001, Durban, 4000 South Africa

**Keywords:** Plant Economic Spectrum, Legumes, Rhizobia, Inoculation, Light availability, Nitrogen fixation, Microbial diversity

## Abstract

Legume–rhizobia symbiosis are fundamental drivers of nitrogen cycling and plant performance, yet their role in facilitating species strategies along the Plant Economic Spectrum (PES) remains insufficiently understood. We conducted a field experiment with four legume species subjected to light and shade treatments, with and without rhizobial inoculation, to assess plant survival, biomass accumulation, nodulation, nitrogen acquisition, and isotopic signatures, alongside microbial community diversity and shifts in vegetation composition. Results demonstrate that inoculation significantly enhanced survival, growth rates, nitrogen accumulation, and nodulation across species, particularly under light conditions, indicating that microbial symbiosis promotes acquisitive strategies within the PES framework. Contrary, shaded environments consistently favoured higher survival and root allocation but reduced growth, nodulation, and nitrogen fixation, reflecting more conservative resource-use strategies. Species-specific responses revealed differential PES positioning: *Trifolium repens* L. exhibited high acquisitive capacity under light, while *Coronilla juncea* L. showed poor survival and growth under both conditions, highlighting the interaction between phylogenetic identity and resource availability. Additionally, δ¹⁵N and %Ndfa values confirmed that inoculation increased nitrogen fixation efficiency, whereas microbial diversity analyses indicated strong shifts in soil bacterial communities associated with inoculated plants, suggesting feedback between symbiosis and soil microbiota. These findings support two main hypotheses: (i) rhizobial inoculation acts as a biotic driver promoting acquisitive strategies by enhancing resource acquisition and growth efficiency, and (ii) light availability serves as an abiotic axis that modulates species positions along the PES continuum. Together, our study provides novel evidence that both microbial interactions and resource availability jointly determine legume strategies within the PES.

**Supplementary Information:**

The online version contains supplementary material available at 10.1007/s00248-026-02777-4.

## Introduction

 Mediterranean-type ecosystems are characterized by seasonal droughts and nutrient-poor soils that accelerate soil degradation. In these environments, legumes play a pivotal role in maintaining ecosystem stability and productivity [1]. Their ability to fix atmospheric nitrogen (N_2_) through symbiosis with rhizobia enriches the soil, enhancing nutrient cycling and supporting plant communities [2]. Light availability in these ecosystems is highly variable, influenced by factors such as canopy cover, topography, and seasonal changes. Light availability is a fundamental determinant of plant performance, influencing photosynthesis, biomass allocation, root architecture, and nutrient acquisition [3, 4]. For legumes, this influence is compounded by their symbiotic relationship with N fixing rhizobia. This mutualism can reduce the need for soil N and enhance plant nutrition. However, the extent to which legumes invest in this symbiosis and how it affects their performance is not only influenced by N availability but also by the plant’s ecological origin and adaptation to light conditions.

Species native to shaded environments, such as *Trifolium repens* L. and *Vicia sativa* L., have evolved strategies that maximize efficiency under low-light conditions, often by increasing below-ground resource allocation when exposed to higher light intensities [5]. In contrast, species adapted to well-lit habitats, such as *Ornithopus compressus* L. and *Coronilla juncea* L., typically allocate more resources to aboveground biomass when light is abundant, exhibiting less plasticity in root architecture and exudate production under varying light conditions [3, 4, 6].

The “Plant Economics Spectrum” theory suggests that plants allocate resources between growth and defence strategies based on environmental conditions [7, 8]. In the context of legumes, this theory suggests that light availability influences how plants balance carbon allocation between photosynthetic activity and symbiotic N fixation. Under low light conditions, legumes may prioritize carbon (C) allocation to support rhizobial symbiosis, enhancing N fixation. Contrary, under high light conditions, they might allocate more C to aboveground growth, optimizing light capture and biomass production [4, 6].

Rhizobial inoculation can modulate these strategies by enhancing N acquisition through biological N fixation (BNF). Inoculated plants often exhibit lower δ¹⁵N values, indicating a greater reliance on atmospheric nitrogen [9, 10]. This shift can alter the balance between aboveground and belowground biomass, enzymatic activities, and root exudate profiles, reflecting the plant’s adaptive responses to light availability [11, 12]. In a previous study under gnotobiotic conditions we [4, 13] found that rhizobial inoculation modulates nutrient acquisition and biomass allocation in legumes exposed to contrasting light regimes, revealing strategies linked to the ecological history of each species. Shade origin species such as *Trifolium repen*s and *Vicia sativa* increased root complexity, nodule production, and root exudation under high irradiance, reflecting a mining strategy that redirects resources belowground to secure nutrients and sustain symbiosis. In contrast, sun adapted *Ornithopus compressus* and *Coronilla juncea* showed much weaker shifts in root traits and instead channelled additional energy into aboveground growth when light was abundant, maximizing photosynthetic returns. Together, these patterns show that rhizobial inoculation enhances N acquisition while reinforcing complementary strategies—belowground investment in shade adapted legumes and biomass expansion in sun adapted ones—that shape performance and rhizosphere function under variable irradiance.

The question that follows is whether plants in natural environments behave similarly to those observed under controlled conditions. In the present experiment, we explored how plants originating from shaded environments (*Trifolium repens* and *Vicia sativa* ) acquire available N and how this translates into biomass production and survival when exposed to high light intensity in a natural setting. In contrast to [14] we aimed to verify that plants originating from well-illuminated environments (*Ornithopus compressus* and *Coronilla juncea* ) will not display a significant increase in biological N fixation under high light intensity in nature but would instead allocate more resources to aerial biomass production.

We further hypothesize that enhanced plant performance of the above species would generate broader environmental benefits, including increased soil microbial diversity, improved soil fertility and greater plant richness. To test these hypotheses, we established field experiments with the four legume species either inoculated or not, under two irradiance regimes, and plant communities were analysed during five years of growth. Understanding how light regimes interact with rhizobial symbiosis to shape legume performance can inform strategies to optimize nutrient uptake, biomass production, and rhizosphere health, particularly under changing environmental conditions.

## Materials and Methods

### Experimental Site

The study was carried out at a privately owned agricultural land near Badajoz in Extremadura (Spain) (38°88′8.99″ N; −7°0′43.3″ W). The farm was fallow, and no treatments had been applied to the soil prior to the experiment, although the site has a long history of cultivation with chemical fertilizers and pesticides. The surrounding region has a typical Mediterranean climate, characterized by cold winters and very hot, dry summers. Most precipitation occurs in spring and autumn (Table S1).

The bedrock is siliceous, with soils averaging a pH of 6.5 and a texture composed of 44.3% sand, 23.6% clay, and 32.1% silt. Additional soil properties included total C of 8.3 g kg⁻¹, total N of 0.81 g kg⁻¹, total phosphorus (P) of 68 ppm, and total potassium (K) of 3.26 meq 100 g⁻¹. At the start of the study, the herbaceous layer consisted of a relatively poor mixture of annual monocotyledonous and dicotyledonous species (Table S2).

### Biological Material

Seeds of *Trifolium repens*, *Vicia sativa*, *Ornithopus compressus* and *Coronilla juncea* were collected from plants growing in natural populations in the surroundings of the campus of the University of Extremadura, in landfills close to the experimental site. Seeds were placed in brown envelopes and stored at 21 ± 1 °C before the experiments were conducted.

Two of the chosen species, *C. juncea* and *O. compressus*, have their natural range of distribution in well-irradiated areas, whereas the other two, *T. repens* and *V. sativa*, are usually found in shaded areas. *Vicia sativa* L. and *O. compressus* L. are annual herbs while *C. juncea* and *T. repens* are perennials, the former being a small shrub and the later an annual. The four species are native to the Mediterranean region and could be considered as pastureland plants or alternative fodder, under the current scenario of climate change.

### Field Experiment

On 4 March 2019, four 2 × 2 m sampling plots were established for each plant species under a split‑plot design, with shaded and full‑sunlight plots positioned 2 m apart. For every species, experimental treatments followed a 2 × 2 factorial structure (light vs. shade; inoculated vs. non‑inoculated), with four replicate quadrats per treatment combination, resulting in a total of 16 plots. Shade was provided using a 3‑mm black mesh net. Each plot was sown with 3000 sterile seeds of the corresponding species, and seedlings were monitored for emergence. One month after sowing annual species and two months after sowing perennials, plots were thinned to a final density of 100 seedlings per quadrat, except for *C. juncea*, for which only 70 seedlings were available. Half of the quadrats received the appropriate rhizobial strain, whereas the rest remained non‑inoculated to serve as controls. Prior to sowing, all seeds were surface‑sterilized by immersion in a 0.5–1% sodium hypochlorite solution for two minutes, followed by ten rinses with sterile distilled water. Seeds were then briefly immersed in 70% ethanol and again rinsed ten times with sterile distilled water.

The bacterial inoculants were selected based on documented host specificity and availability in our collection: *Rhizobium leguminosarum* USDA2370T for *V. sativa* and *C. juncea*, *Rhizobium leguminosarum* and *R. leguminosarum* subsp. *trifolii* ATCC1440 for *T. repens.*, and *Bradyrhizobium canariensis WSM1253* for *O. compressus*. Inoculants consisted of a dense suspension in YMA broth with an average cell density of 9 × 10⁹ cells mL⁻¹, applied at 330 mL per plant. To prevent cross-contamination, inoculated plots, whether in the sun or shade, were positioned 2 m away from the corresponding non-inoculated plots. Seedlings were watered fortnightly for the first six months after transplanting and then once a month for the following 12 months to support their establishment. Watering was applied manually, with 2 L of sterile distilled water per plant.

An additional control plot was established at the northern part of the experimental site.

### Data Collection

Soil moisture, temperature, and electrical conductivity were measured using capacitance-based soil sensors (5TE, METER Group Inc.) and validated with a TDR probe (CS650, Campbell Scientific). Sensors were installed at 10 (± 1) cm depth from the surface and logged automatically throughout the study.

Photosynthetically active radiation (PAR) was measured for each species under sunny and shaded light treatments using a handheld quantum sensor (LI‑190R Quantum Sensor, LI‑COR Biosciences) connected to a portable light meter. Measurements were taken at canopy height to capture the effective PAR (E PAR) experienced by the leaves. For each plant, readings were collected between 11:00 and 14:00, corresponding to peak daylight hours, and averaged to obtain a single E PAR value per sampling event. Measurements were conducted at the time that survivorship was recorded (twice a year) throughout the study period to account for seasonal variation in incident light.

### Biological Data Collection

Short-term plant performance was assessed after one growing season by measuring individuals of the annual species, while long-term performance was evaluated in the perennial species. Plant survivorship was monitored every six months, except in 2020, when only the autumn survey was conducted due to the pandemic. Each spring (March–May), herbaceous species richness and diversity were recorded in each plot (Table S2).

During the first growing season, after 70 days of growth, 15 seedlings per species and treatment were harvested to calculate relative growth rate (RGR). The final harvest was conducted after a total of 123 days in the field. Below-ground allocation represents the fraction of newly formed biomass allocated to roots and nodules over the growth period.

This was calculated according to [15]:

1 *df/dt=RGR(∂-Br/Bt)*

where RGR is the relative growth rate (mg.g^− 1^ ·day^− 1^) and ð is the fraction of new biomass gained during the growth period. Br/Bt is the root weight ratio, which is based on the total plant biomass (Bt) and root biomass (Br).

All plant biomass was dried in an oven at 70 °C until constant weight. Dried plant material (shoot and root) was then weighted, ground and analyzed for N content. Additionally, a separate subsample was sent to the University of Lisbon for isotopic analyses alongside with soil samples.

After 5 years of growth, soil samples were randomly collected from the surface to 10 cm deep from each plot and thoroughly mixed. In each plot five samples were taken to make a composite sample for each plot type (in total nine samples). Soils were analysed following routine methods [16].

### Rhizobial Diversity in Nodules and Soils

Rhizobia were counted in soils within the study plots and in root nodules from inoculated and non-inoculated seedlings of the four species grown in the field.

Rhizobial diversity from root nodules of the four species (I and NI) grown in the field under shade and full light, was assessed by excising 15–38 nodules per species from random plants in each study plot. Every single colony grown between three and 14 days was analysed by BoxAir polymerase chain reaction (PCR). PCR amplification was performed using the primer pair 63 F (5′‑CAGGCCTAACACATGCAAGTC‑3′) and 1387R (5′‑GGGCGGTGTGTACAAGGC‑3′). Each 25 µL reaction mixture consisted of 12.5 µL Sigma-Aldrich KOD Hot Start Master Mix containing 0.5 µL of each primer, 3 µL of a diluted suspension prepared from a single bacterial colony, and 8.5 µL of sterile Milli‑Q water. Reactions were run on a T100 Thermal Cycler (Bio‑Rad, USA) under the following conditions: an initial denaturation step at 94 °C for 2 min, followed by 30 amplification cycles comprising denaturation at 92 °C for 30 s, annealing at 56 °C for 45 s, and extension at 75 °C for 45 s. A final elongation at 75 °C for 10 min completed the program. PCR products were separated on 1% (w/v) SeaKem agarose gels, stained with 6× Orange LD (3 µL per sample), and visualized under UV illumination. Amplicons of the expected size (~ 1324 bp) were excised and submitted for Sanger sequencing at the Genetics Department of The Complutense University (Madrid). Resulting sequences were compared against the GenBank database using BLASTn (NCBI; http://blast.ncbi.nlm.nih.gov/Blast.cgi; accessed 20‑08‑2023). *Bradyrhizobiun elkanii* was used as a control strain.

Rhizobia were extracted from soils collected both before planting (2019) and after plant growth (2024) to quantify changes in the soil rhizobial assemblage. Soil samples were homogenized and serially diluted following standard procedures for rhizobial isolation from soil suspensions [17–19]. Briefly, 10 g of fresh soil were added to 90 mL of sterile physiological saline (0.85% NaCl) solution, shaken for 30 min, and serial tenfold dilutions (10⁻¹–10⁻⁶) were prepared. Aliquots (100 µL) of each dilution were plated onto yeast extract**–**mannitol agar (YEMA) supplemented with Congo red. To reduce growth of non-rhizobial microorganisms, plates were also prepared using semi-selective media MGYF (Mannitol–Glucose–Yeast extract–Fructose agar) and MNBP (medium with Nalidixic acid, Benomyl, Pentachloronitrobenzene and Penicillin G), which include antibiotics and antifungal agents that suppress Gram-positive bacteria, fungi, and actinomycetes while permitting growth of both fast- and slow-growing rhizobia [17, 18]. Plates were incubated at 28–30 °C for 3–7 days [20], and colonies with morphological features typical of rhizobia (smooth, mucilaginous, white to translucent) were sub-cultured to fresh YEMA plates for purification and further analyses by Box Air polymerase chain reaction as per rhizobia from nodules.

### Bulk Soil Bacterial Diversity

Only plots of *T. repens*. were used for this study as it was one of the two perennial species in the experiment. Plants of *T. repens* were chosen as they grew taller and look healthier than those of *C. juncea*, which also had few surviving individuals per plot.

For each treatment, composite soil samples were obtained in April 2024 from the upper 10 cm after removing surface debris. Total DNA was extracted from 250 mg of homogenized soil using the PowerSoil DNA Isolation Kit (Qiagen, Germany) following the manufacturer’s protocol. DNA concentration and purity were assessed with a NanoDrop 1000 spectrophotometer (Thermo Scientific). Bacterial community composition was characterized by 16 S rRNA gene amplicon sequencing targeting the V3–V4 region using primers 341 F/806R. Libraries were prepared according to Illumina specifications and sequenced on an Illumina MiSeq platform (2 × 300 bp) at Macrogen Inc. (Seoul, South Korea). Raw reads were quality-filtered, denoised and classified using the SILVA 138 database. Relative abundances of bacterial phyla were calculated for each sample and averaged per treatment. Control 2019 corresponds to soil collected at the same plots before the experiment began and Control 2024 corresponds to soil collected in 2024 in the control plot. We concentrated on the composition and diversity of bacterial communities, to simplify the study and because bacteria generally dominated soil communities [21, 22]. Note that this analysis represents 16 S rRNA gene amplicon sequencing rather than whole-genome metagenomics. As such, taxonomic resolution is limited to the amplicon region and cannot provide strain-level identification or functional gene inference.

### Data Analyses

Three and two-way analysis of variance (ANOVA) and Tukey’s tests were used to compare data on environmental variables, nodules and total biomass production for inoculated and non-inoculated plants grown in the well illuminated and shaded plots.

All data were tested for normality beforehand and arc-sin transformed when needed. For all host plant– rhizobia interactions, we also examined nodule formation, and a two-way ANOVA was conducted to search for differences between treatments regarding infectiveness of both plants and strains.

Percentage of survivorship in the field after 24 months was analysed by simple binary logistic regression, where survival after summer was the dependent variable and species and inoculation were the predictor factors. Because we detected a very significant species–inoculation interaction (χ2 = 45·5; *p* < 0·001), and exploratory analysis showed differences among species, we made separate one-factor (inoculation) logistic regression for each species. Sample size of treatments was 26–35 seedlings due to different mortality following transplanting. Analyses were conducted with the statistical package SPSS v25·0 [23], and differences were set significant at *p* < 0·05. Where the ANOVA showed significant differences between treatments, a Tuckey’s post hoc test was used to separate the means. Where a two-way ANOVA showed significant effects at 95% (*p* < 0.05), Fisher’s least significant differences were determined using a one-way ANOVA .

Data from vegetation sampling were used to calculate per plot and sampling date: (i) herbaceous richness as the total number of species; (ii) herbaceous diversity as the Shannon–Wiener index [24] using the richness and frequency of each species. Biomass was calculated using the dried weight of the collected aerial-plant material and expressed as g/m^2^. Total above-ground biomass, estimates of species richness and diversity under the two light regimes (I and NI) per plot for the four species, were compared using two-way nested ANOVA. The effect of the plants on soil fertility and herbaceous richness, diversity, cover and biomass were studied by Student t-test for overall comparisons among data obtained in each plot type.

For rhizobial populations in the soil, values of most probable numbers were calculated from the number of nodulated test plants at each dilution level, by reference to frequency tables. It gives a range of 1 to 11 770 organisms per ml of suspension extracted from 10 g of soil [25]. Shannon’s diversity index [24] was calculated for each combination of plant species and treatment in regard to numbers of rhizobial strains identified from nodules collected from plants grown in the inoculated and non-inoculated plots in the field.

## Results

### Environmental Variables

Soil physical variables showed clear treatment differences. Soil temperature was slightly higher under full light than under shade, while variation among species was minimal (Figure S1). A three-way ANOVA (Species × Light × Inoculation) detected a significant effect of Light (F₁,₁₅₂= 14.13, *p* = 0.00024), whereas species, inoculation, and all interaction terms were not significant (all *p* ≥ 0.95). Tukey post hoc comparisons confirmed the absence of significant pairwise differences among light and inoculation combinations within any species (*p* ≥ 0.56).

Electrical conductivity also differed between light treatments, with consistently higher values under full sun (Figure S1). The three-way ANOVA detected significant main effects of light (F₁,₁₅₂ = 220.04, *p* < 10⁻³⁰) and inoculation (F₁,₁₅₂= 9.02, *p* = 0.00314), while species and all interaction terms were non-significant (*p* ≥ 0.48). Tukey post hoc tests showed that EC values were significantly higher under sunny than shaded conditions for most species (adjusted *p* < 0.05), while inoculated treatments exhibited small but significant increases relative to non-inoculated ones where differences occurred. Among species comparisons within each light vs. inoculation level significant differences were detected (*p* ≥ 0.05).

Effective PAR (E PAR) differed strongly between light treatments. A two-way ANOVA detected a highly significant effect of light on E PAR (F₁,₇₄= 132.20, *p* < 0.0001) and a significant species vs. light interaction (F₃,₇₄ = 9.71, *p* < 0.0001), while for the main effect of species no significant differences were detected (*p* = 0.627). Tukey HSD pairwise comparisons showed that, within every species, E PAR under sunny conditions was significantly higher than under shaded conditions (*p* < 0.001), with mean differences of + 615.2 µmol m⁻² s⁻¹ for *T. repens*, + 742.7 for *V. sativa*, + 1712.6 for *O. compressus*, and + 1932.0 for *C. juncea*. Comparisons among species within each light treatment indicated that, under shaded conditions, *T. repens* and *V. sativa* had significantly higher E PAR than *O. compressus* and *C. juncea* (*p* < 0.0001), whereas under sunny conditions only the contrast between *C. juncea* and *T. repens* was significant (*p* = 0.040) (Figure S2).

### Plant Survivorship

**Fig. 1:**
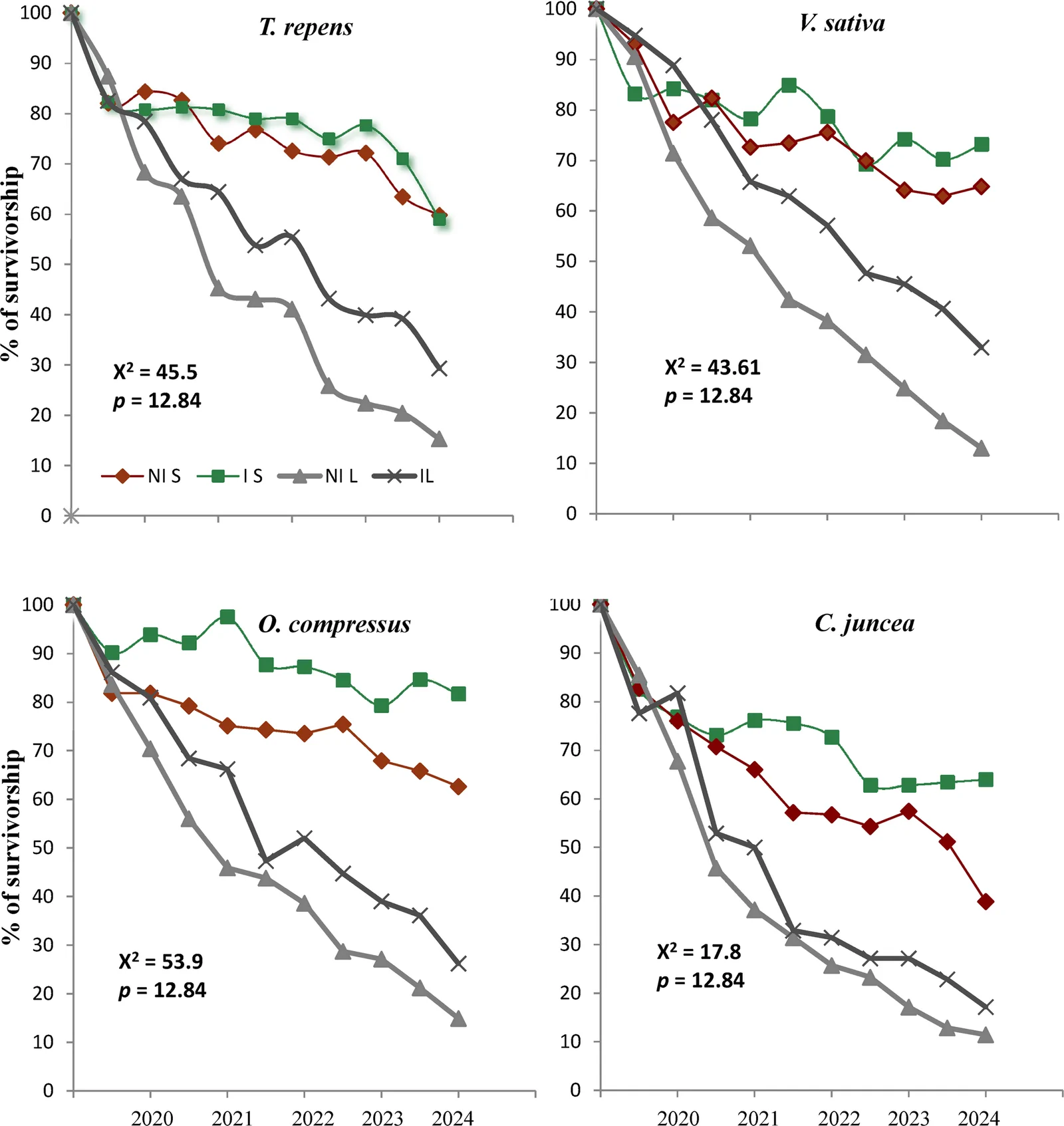
Survivorship of plants of *T. repens*, *V. sativa*, *O. compressus* and *C. juncea* grown in Non-Inoculated Shade (NI S), Inoculated Shade (I S), Non Inoculated Light (NI L) and Inoculated Light (I L). in the field

Survival data after 55 months showed that inoculation enhanced seedling survivorship in all four species. However, the highest survival rates were recorded in plants grown under shade, regardless of inoculation treatment (Fig. 1). Inoculated seedlings of *V. sativa* and *O. compressus* grown in the shade achieved survival rates exceeding 70%, while the lowest survival was observed in *C. juncea* grown under full light (Fig. 1).

### Biomass Production and Gowth Kinetics

**Fig. 2:**
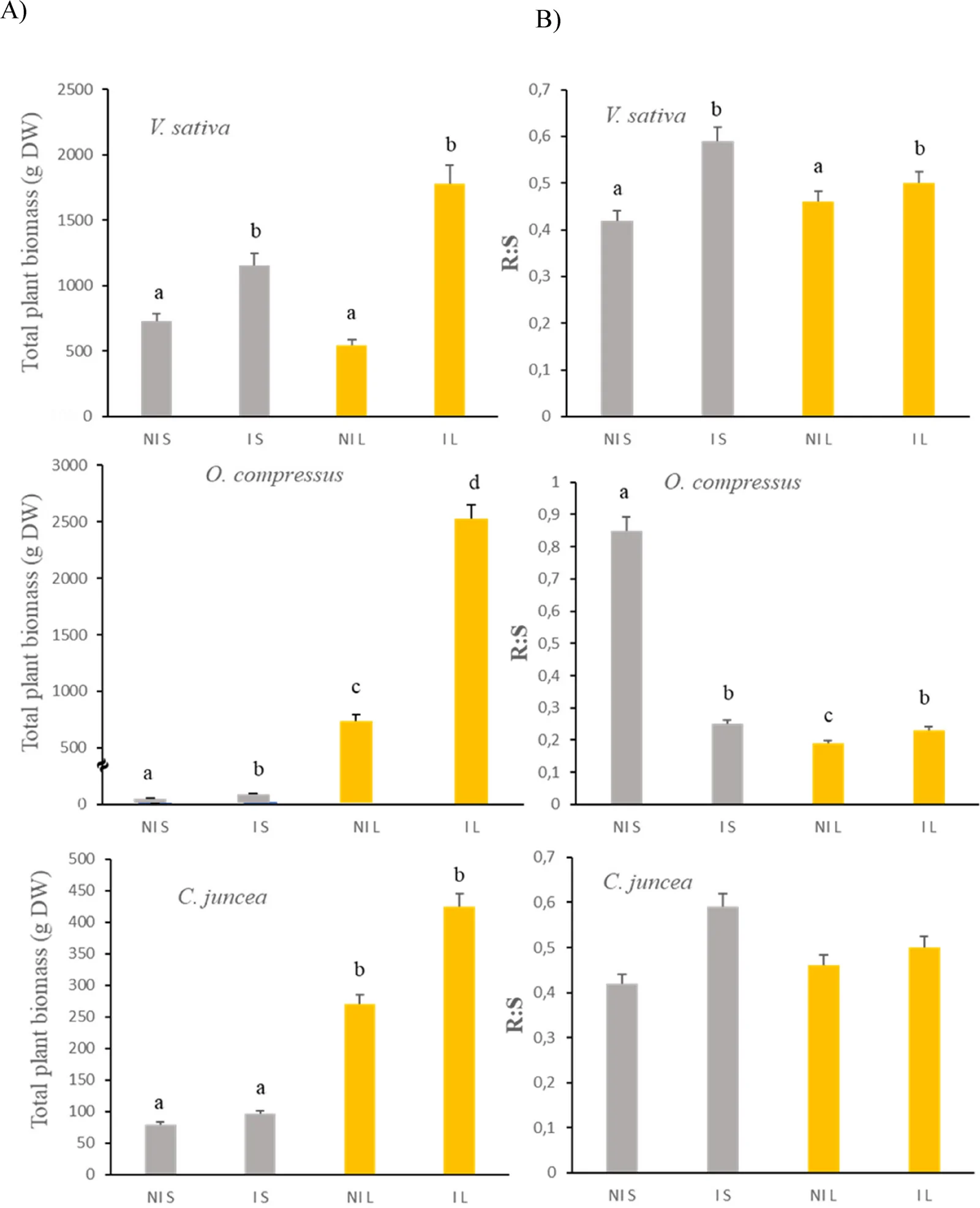
Plant dry weights (**A**) and root : shoot ratio (**B**) of *T. repens*, *V. sativa*, *O. compressus *and *C. juncea* grown in the field under full light or in the shade with and without bacterial inoculation. Values are presented as means (n = 15) with standard error bars and different letters on top of the columns indicate significant differences among treatments using the post hoc Fisher’s LSD, multiple range test (*P* ≤ 0.05). Note different scales

Across the four species, total biomass and biomass allocation between shoots and roots showed marked treatment specific responses that were consistent between the S: R ratios (Fig. 2B) and the below ground allocation coefficients (df/dt) derived from growth analysis (Table 1). In *T. repens*, inoculation generally increased total biomass under both shade and full light (Fig. 2A), and this was accompanied by modest but consistent changes in below ground allocation. Specifically, df/dt values ranged from 8.86 in the non-inoculated light treatment to 46.65 in the non-inoculated shade, while inoculated plants showed intermediate values under both light conditions 198.33 in shade and 176.42 in full light), indicating that inoculation did not substantially alter root allocation in this species.

**Table 1:** Below ground allocation (%) of *T. repens*,*V. sativa*, *O. compressus *and *C. juncea* grown in the field under full light or in the shade with and without bacterial inoculation. Values are presented as means (*n *= 15) with standard deviation. The different letters indicate significant differences among the treatments, where the prime lettering indicates the comparisons between the same organ (*P *≤ 0.05) and the second between plant species

	*T. repens*	*V. sativa*	*O. compressus*	*C. juncea*
**NI S**	46.65±4.34^a^	180.73±13.81^a^	1.75±0.11^a^	16.38±1.49^a^
**I S**	198.33±10.71^b^	284.95±18.29^b^	1.97±0.09^a^	9.34±0.41^a^
**NI L**	8.86±0.61^c^	131.23±9.93^a^	46.39±3.66^b^	37.08±0.41^a^
**IL**	176.42±5.90^b^	386.20±26.49^c^	756.86±40.59^c^	69.48±11.90^b^

In *V. sativa*, which showed one of the strongest improvements in biomass under inoculation—particularly in full light—below ground allocation also increased consistently. df/dt values rose from 180.73 (non-inoculated shade) and 131.23 (non-inoculated light) to 284.95 in inoculated shade and 386.20 in inoculated full light, reflecting a notable reinforcement of root allocation under inoculation that aligns with reduced S: R ratios in the same treatments. In contrast, *O. compressus* exhibited a consistently root dominated strategy under all conditions: S:R ratios were low (Fig. 2B) and df/dt values ranged from very low under non inoculated shade (≈ 0.016) to extremely high under inoculated light (≈ 7.58), indicating a strong shift toward root investment when conditions were favourable. Finally, *C. juncea* showed intermediate patterns, with df/dt values increasing from 0.1638 in non-inoculated shade to 0.6948 under inoculated light. These results demonstrate that species differ markedly in their allocation strategies, and that df/dt values closely parallel S: R ratios, reflecting coherent shifts in carbon allocation to roots in response to inoculation and light availability.

**Fig. 3:**
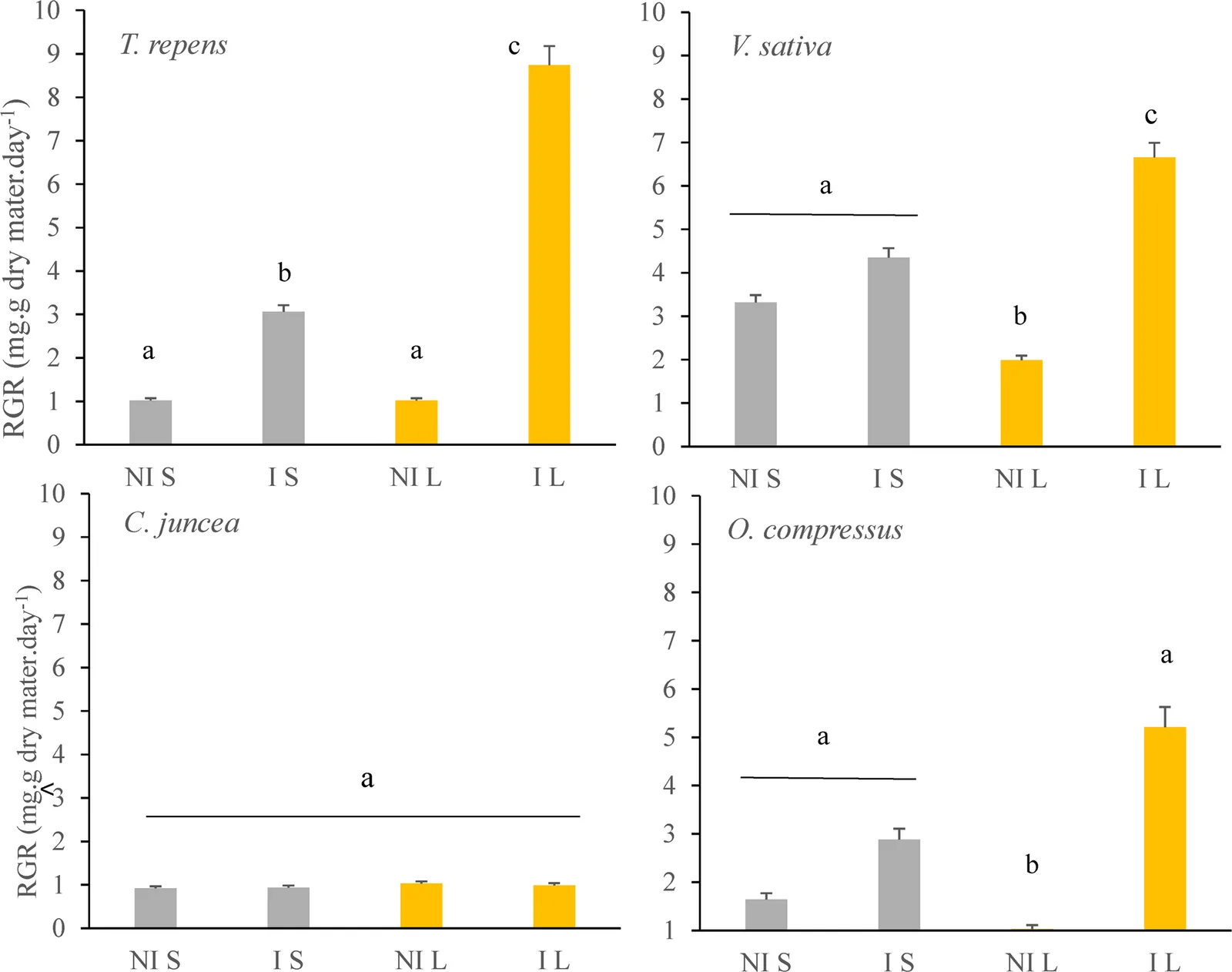
Relative Growth Rate of *T. repens*, *V. sativa*, *O. compressus *and *C. juncea* grown in the field under full light or in the shade with and without bacterial inoculation. Values are presented as means (n = 15) with standard error bars and letters indicate significant differences among treatments using the post hoc Fisher’s LSD, multiple range test (*P* ≤ 0.05)

The relative growth rates (RGRs) for total plant biomass (Fig. 3) were significantly much higher in the plants grown with the inoculation treatment for the four species. The exception was *C. juncea*, for which we did not identify a benefit from either the light or the inoculation treatment (Fig. 3). In general, the relative growth rate in plants from shaded environments was greater than that in plants from well-irradiated ones. The plants of *T. repens*, *V. sativa*, and *O. compressus* reached a significantly greater RGR in the well irradiated plots whereas no consistent pattern was observed in plants of *C. juncea*.

**Fig. 4:**
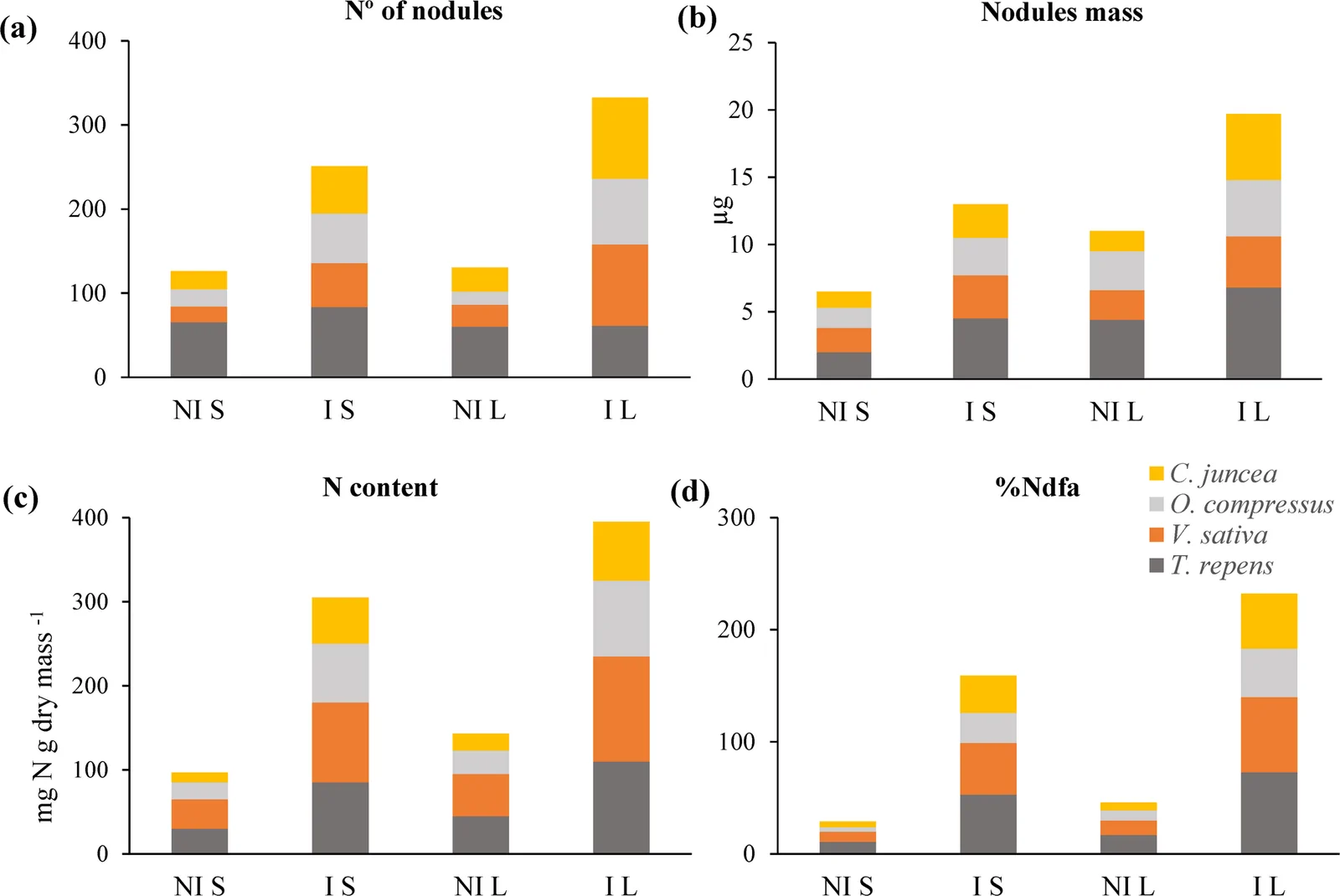
Number of nodules (**a**), nodule biomass (**b**), plant’s N concentration (**c**) and, % of Nitrogen dervied from the atmosphere (%NDFA) in plants of *T. repens*, *V. sativa*, *O. compressus* and *C. juncea* in the field, inoculated and non-inoculated in full light and in the shade (NI S: non-inoculated in the shade; IS: inoculated in the shade; NI L: non-inoculated in the light and; I L: inoculated in the light). The values are the means ± SE of 15 individuals per treatment

Roots of all plants under the four treatments produced nodules (Fig. 4), although plants in the inoculated plots showed a greater number of heavier nodules. There were significant differences related to the light treatments under which the plants grew. The nodulation in the plants grown in the light treatment was significantly (*p* = 0.0023) higher than those grown in the shaded plots, regardless of the species considered, except for *T. repens*, which did not show differences in nodulation between the two levels of radiation. Variations in the nodule number and mass were observed among the legumes originating from light (*C. juncea* and *O. compressus*) and shaded environments (*T. repens* and *V. sativa*) (Fig. 4a and b). In the case of *T. repens*, after five years of field growth, the number of nodules tripled, while their size increased by a factor of 1.5 in inoculated plants. No significant differences were observed in the plants under the remaining treatments.

Nitrogen accumulation in the four species followed a similar pattern, with the highest values observed in the inoculated treatments, particularly in the light plots (Fig. 4c). The two anual species, *V. sativa* and *O. compressus* accumulated the greatest amounts of N, with maximum values recorded in the inoculated+light treatments. This pattern was also reflected in the %Ndfa across all four species and treatments (Fig. 4c and d). In *T. repens*, nitrogen accumulation after five years of field growth increased fourfold, with no significant differences observed among treatments. The %Ndfa also increased, by a factor of 1.5 in non-inoculated plants and by a factor of 4 in inoculated ones.

Isotopic analysis of above-ground plant material resulted in values of δ^15^N very close to or below zero for the four leguminous plants in the inoculated plots. Similarly, values of δ^15^N in plants from non-inoculated plots were very low. The minimum values were observed in the inoculated plants grown in the light, regardless the species, being the minimum for *T. repens*, followed by *V. sativa* and *O. compresuss* (Table 2). In all cases, values of δ^15^N in the legumes was significantly lower than that in the reference plants. The obtained soil δ^15^N values reflect the strong influence of inoculated legumes on soil nitrogen inputs, because the average values decreased from an average of 4.88 ± 0.16‰ in non-inoculated stands to 2.4 ± 0.26‰ in inoculated stands. The lowest values of soil δ^15^N were recorded in stands of inoculated plants grown in the light after 5 years of growth (Fig. 4d).

**Table 2:** Values of δ^15^N values of soils and plants of *T. repens*, *V. sativa*, *O. compressus* and *C. juncea* inoculated and non-inoculated grown in the field in full light and in the shade. Values of the reference plants were estimated in a mixture of plants in the Poaceae family growing next to the target legumes. Values are in ‰. * indicate significant differences with the control plot, after a t-test

	δ^15^*N* (‰)	Soils		δ^15^*N* (‰)	Plants
		1 year	5 years	1 year	5 years
**Control**	6.6	6.9	5.5	6.6	6.3
***T. repens***					
NI S		5.8	3.3	2.3	1.1
I S		4.4	2.3	1.8	0.6*
NI L		4.3	3.8	1.2	0.1
I L		3.9	1.3*	0.4	−0.01*
***V. sativa***					
NI S		4.8	2.1	3.5	2.3
I S		2.4	1.6	2.2	0.9*
NI L			2.5	4.1	2.8
I L			1.5*	0.7	0.3*
***O. compressus***					
NI S		5.5	5.0	3.5	2.1
I S		3.8	1.2*	1.8	0.2*
NI L		4.6	3.9	2.7	1.2
I L		3.4	1.3*	0.9	−0.2*
***C. juncea***					
NI S		5.9	4.7	4.0	4.7
I S		4.3	2.6	2.7	1.0*
NI L		5.1	3.9	3.3	3.6
I L		3.3	1.8*	0.9	0.1*

As for the most probable numbers of soil rhizobia, inoculated *T. repens* and *V. sativa* under shade showed higher values of soil rhizobia in 2021 (Table 3) as compared to non-inoculated ones, whereas in 2024 inoculated *T. repens* showed lower values under shade, and under light in both years, although not always significant. In *O. compressus* and *C. juncea* lower values were observed in inoculated plants under shade and well-illuminated environments in both years.

**Table 3:** Most probable numbers (MPN) of soil rhizobia [log10 (MPN + 1)] in five experimental plots, detected by plant infection test using *T. repens*, *V. sativa*, *O. compressus* and *C. juncea*. at two different times: before legumes growth in the plots (2019) and after five years of legume growth in the soils (2024). Data are menas of three soil replicates per site and standar deviations are in parenthesis. (I: Inoculated; NI: Non-Inoculated; S: Shade; L: Light). Different letters next to the numbers indicate significant differences after a two-way ANOVA

2021	T. repens	V. sativa	O. compressus	C. juncea	Control
NI S	0.46 (0.21) ^a^	0.15 (0.01)^a^	3.09 (0.86) ^a^	3.03(0.23) ^a^	0.24 (0.06) ^a^
I S	0.64 (0.11) ^a^	1.06 (0.55) ^b^	2.85 (0.83) ^a^	2.64 (0.37) ^a^	2.03 (0.73) ^b^
NI L	1.71 (0.27) ^b^	1.61 (0.70) ^b^	3.46 (0.59) ^a^	10.94 (0.89) ^b^	0.16 (0.03) ^a^
I L	0.73 (0.12) ^a^	1.11 (0.19) ^b^	3.14 (0.65) ^a^	2.13 (0.67) ^a^	0.74 (0.27) ^a^
**2024**	*T. repens*	*V. sativa*	*O. compressus*	*C. juncea*	*Control*
NI S	9.72 (1.19) ^a^	7.62 (0.96) ^a^	7.56 (0.91) ^a^	6.24 (0,56) ^a^	3.91 (0.95) ^a^
I S	8.58 (1.26) ^a^	7.62 (1.29) ^a^	4.36 (0.96) ^b^	2.36 (0.18) ^b^	2.41 (0.61) ^a^
NI L	11.10(1.63) ^b^	11.77 (0.77) ^b^	7.12 (0.95) ^a^	4.31 (0.67) ^b^	4.81 (0.25) ^a^
I L	10.11 (1.39) ^b^	6.96 (0.47) ^a^	3.48 (0.84) ^b^	0.65 (0.28) ^b^	3.79 (0.74) ^a^

### Accompanying Vegetation

**Fig. 5:**
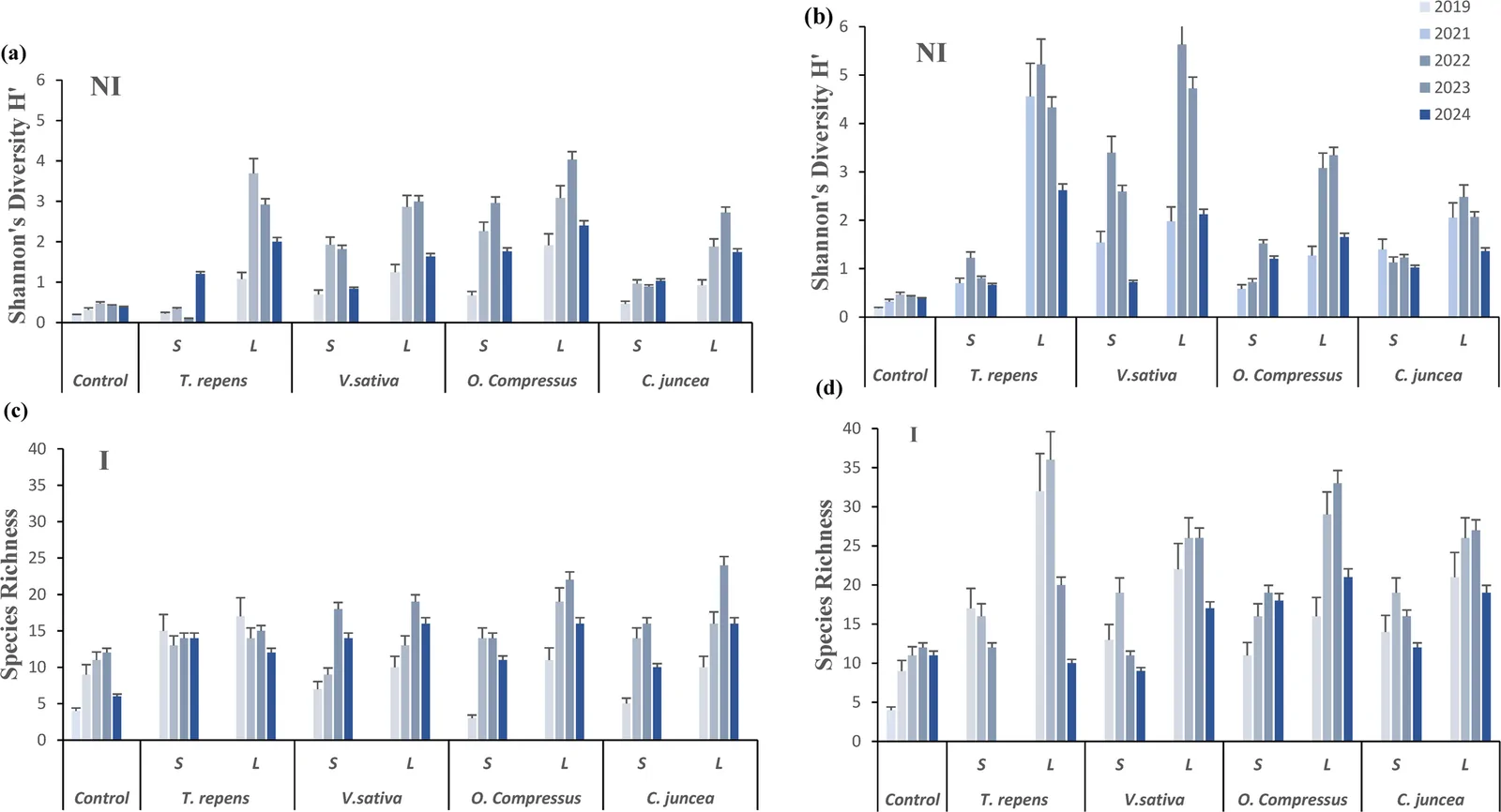
Shannon’s diversity index (H’) and species Richness in plot where plants of *T. repens*, *V. sativa*, *O. compressus* and *C. juncea* grew in the field, inoculated (**a** and **b**) and non-inoculated (c y d) both in full light and in the shade NI: non-inoculated; I: inoculated; S: shade; L: light. The values are the means± SE of 4 squares per treatment. Control refers to samples taken in 2019 before the onset of the experiment

Species diversity and richness varied notably across years, treatments, and light conditions. In the non-inoculated (NI) treatments, the Shannon’s index values increased from 2021 to 2023 across all species (Fig. 5), with V. *sativa* and C. *juncea* showing the highest values under shade (S) in 2023 (2.99 and 4.03, respectively), while light (L) conditions generally yielded lower values. Inoculated (I) treatmentsconsistently enhanced diversity, particularly in V. *sativa* (S) plots, which peaked at 5.63 in 2022, and O. *compressus* (S), reaching 4.72 in 2023.T. *repens* showed moderate increases under inoculation, with biomass under shade rising from 0.698 in 2021 to 1.22 in 2022.Richness followed a similar trend, with inoculated plots exhibiting higher values than NI counterparts. By 2023,C. *juncea *(S) reached arichness of 33, and V. *sativa *(S) attained 26, both under inoculation. Light conditions generally supported lower richness, although T. *repens *(L) maintained relatively high values (17 in 2021 and 16 in 2022). Overall, inoculation and shade conditions synergistically promoted both biomass and richness, with peak values observed in 2022–2023 across most species.

### Rhizobial Diversity in Nodules and Soils

**Fig. 6:**
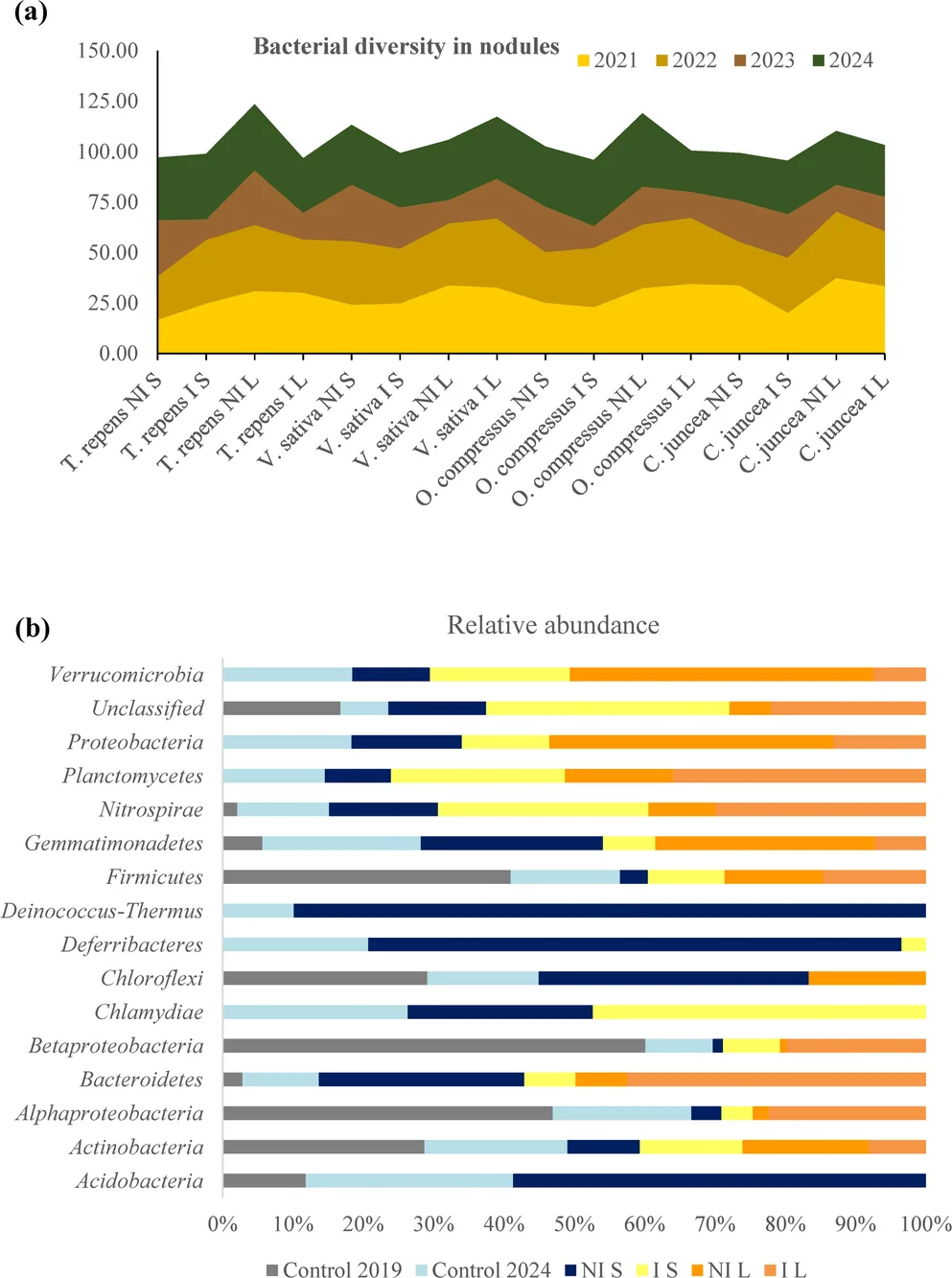
Microbial diversity in nodules: (**a**) in plants of *T. repens*, *V. sativa*, *O. compressus* and *C. juncea*; and (**b**) in soils of *T. repens*, in the field, inoculated and non-inoculated both in full light and in the shade NI S: non-inoculated in the shade; IS: inoculated in the shade; NI L: non-inoculated in the light and; I L: inoculated in the light). The values are the means ± SE of 15 individuals per treatment. Control refers to samples taken in 2019 before the onset of the experimentnull

Although we aim at identifying rhizobial strains in the nodules, we also detected other taxa (Fig. 6**b**, Table S3). Microbial abundance in nodules varied significantly across legume species, treatments, and years. Overall, plants grown under light generally exhibited higher microbial diversity, particularly in *T. repens* and *C. juncea*, which reached peak values of 30.99 and 37.44 respectively in 2021. In 2022, diversity values under light remained high or even increased in several species, particularly in inoculated treatments of *V. sativa* and *T. repens*. Temporal trends showed an overall increase in nodule microbial diversity from 2021 to 2024, although with species and treatment specific fluctuations. Notably, *T. repens* and *O. compressus* displayed a marked rise over time, whereas *C. juncea* and *V. sativa* exhibited more variable patterns across years.

### Soil Microbial Diversity

Illumina MiSeq 16 S rRNA amplicon sequencing performed in soils of *T. repens* for all the treatments and years revealed clear differences in microbial community composition across treatments. The relative abundance of soil bacterial taxa varied markedly across treatments (data only for 2024) compared to the control samples collected in 2019 (Fig. 6). *Acidobacteria*, virtually absent in all 2024 treatments, showed a notable increase under non-inoculated shade (NI S: 8.95%). *Alphaproteobacteria* and *Betaproteobacteria*, the most abundant phyla in control soils (28.6% and 12.56%, respectively), were reduced under all treatments, with a partial recovery of *Alphaproteobacteria* under inoculated light (I L: 13.6%). *Bacteroidetes* and *Firmicutes* exhibited increased abundance under light and inoculated conditions, particularly *Bacteroidetes* in I L (11.75%) and *Firmicutes *in NI L and I L (2.99% and 3.09%, respectively. Notably, *Proteobacteria* as a broader group peaked under NI L (57.37%), suggesting enhanced microbial activity or recruitment in non-inoculated light-exposed soils. Several minor phyla, including *Chlamydiae*, *Deinococcus-Thermus*, and *Deferribacteres*, were detected mainly in shaded treatments. The proportion of unclassified bacteria was highest under inoculated shade (I S: 55.94%), reflecting either novel taxa or limitations in taxonomic resolution under those conditions. Overall, these results high light the strong influence of light and inoculation on soil microbial community structure, with clear shifts in taxonomic composition relative to baseline conditions.

## Discussion

Plant performance across species reflected strong interactions between light availability and rhizobial inoculation, with shade consistently enhancing survival but reducing growth and nodulation. Interpretation of the shade treatment must also consider the broader microclimatic changes induced by the shade nets. Our measurements show that shading reduced effective PAR most strongly but also produced modest decreases in soil temperature and electrical conductivity (Figure S1). These shifts indicate that the shade treatment altered not only irradiance but also evaporative and energetic load, consistent with previous reports that shade nets simultaneously modify multiple microclimatic axes [3, 5]. Therefore, the observed increases in survival and changes in biomass allocation under shade likely reflect a combination of reduced light availability and reduced thermal and water stress. This multidimensional response aligns with ecophysiological expectations for Mediterranean environments, where high irradiance and evaporative demand impose strong constraints on early seedling establishment [1].

Across species, inoculation consistently enhanced growth, N acquisition, and nodulation, particularly under full light, supporting the interpretation that microbial symbiosis promotes acquisitive strategies when C is abundant [9, 11, 12, 26]. In contrast, shaded plants exhibited higher survival and root allocation but lower N fixation, consistent with more conservative resource‑use strategies described in the Plant Economic Spectrum [7, 8]. These patterns align with previous findings that legumes from shaded environments increase below‑ground investment under high irradiance [4], whereas sun‑adapted species prioritize above‑ground growth when light is abundant [6]. By integrating these responses with the microclimatic context described above, our results support the interpretation in which both light availability and associated thermal–hydric conditions jointly shape legume performance and PES positioning in the field.

Inoculation enhanced total biomass in all four species, particularly under full light, where resource availability allowed plants to exploit the benefits of symbiosis. This shift toward an acquisitive strategy was most evident in *T. repens*, *V. sativa*, and *O. compressus*, which displayed greater relative growth rates (RGRs) under light and inoculation as previously seen under gnotobiotic conditions [4]. Shade, in contrast, induced higher root allocation, particularly in the species originating from shaded environment. This is consistent with conservative strategies aimed at optimizing belowground foraging and persistence under resource limitation [6, 27] and with previous observations under controlled growth conditions [3]. The exception of *C. juncea*, which did not benefit from inoculation or light, underscores that species adapted to high-irradiance habitats may exhibit more fixed functional strategies, with reduced plasticity along the PES continuum.

Nodulation occurred in all plants regardless the treatment. This is due to the natural presence of nodulating bacteria in the soil. Nevertheless, plants responded strongly to inoculation and light, with greater numbers and mass of nodules in light-grown inoculated plants. Previous studies have demonstrated that *T. repens* reduced total nodules biomass under shade growing conditions [6]. In a previous study [4], we observed the same pattern of nodule’s mass reduction in *T. repens*,* V. sativa*,* O. compressus* and *C. juncea* in low radiation treatments. These observations suggest a trade-off: nodules are costly in terms of C, and when light (thus C supply) is limited, plants may reduce nodulation or invest less in nodule size or number to avoid carbon debt [26]. In full light + inoculation treatments, plants had sufficient C resources to support greater nodule number and mass, enhancing N fixation and pushing trait expression toward rapid growth. In fact, N accumulation and %Ndfa followed the same pattern, with the highest values in inoculated plants under light. Annual species such as *V. sativa* and *O. compressus* exemplified acquisitive PES traits by maximizing N capture and growth in these conditions. Conversely, in *T. repens*, nodulation and N accumulation increased more gradually over five years, reflecting a conservative strategy in which long-lived perennials spread symbiotic benefits over time [12].

Isotopic data provide further evidence that inoculation and light synergistically shift plants toward acquisitive strategies. The lowest ^δ^15 N values in inoculated plants grown in light indicate high reliance on atmospheric N₂ fixation and efficient symbiosis as reported before [12, 13]. These effects extended to the soil, where reduced ^δ^15 N values under inoculated light conditions reflect sustained N enrichment from biological N fixation (BNF). In PES terms, this illustrates how acquisitive strategies not only enhance plant performance but also drive ecosystem-level nutrient cycling, potentially reinforcing positive feedback for accompanying vegetation.

Microbial diversity within nodules varied across species, treatments, and years, with higher diversity in non-inoculated plants grown under light and lower diversity in shaded inoculated treatments. The multi‑year nodule sequencing dataset (Table S3) further supports the conclusion that nodules in field conditions host diverse bacterial assemblages. Across all species and treatments, nodules consistently contained the expected rhizobial symbionts (e.g., *Rhizobium leguminosarum*, *Bradyrhizobium canariensis*), but also a range of non‑rhizobial endophytes such as *Pseudomonas*, *Bacillus*, *Serratia*, *Stenotrophomonas* and *Microbacterium*. These patterns were stable across years and occurred in both inoculated and non‑inoculated plants, indicating that inoculation did not eliminate native symbionts nor prevent co‑colonization by other bacterial taxa. This mixed occupancy is typical of field nodules and reflects the competitive and ecological complexity of natural soils. These patterns suggests that resource availability and partner selection strongly influence nodule colonization. Under high-resource conditions (light), plants may exert stronger selection for efficient symbionts, favouring dominance of a few competitive rhizobial strains and resulting in lower overall microbial diversity. In contrast, under low-resource conditions (shade) or in the absence of inoculation, selection pressure appears weaker, allowing a broader range of microbial taxa to colonize the nodules [28]. Microbial competition likely reinforces these dynamics: when resources are abundant, highly competitive rhizobia can outcompete other taxa, whereas in resource-limited environments competitive exclusion is reduced, enabling coexistence of a more diverse microbial community. Together, these results highlight how environmental conditions and microbial interactions jointly shape the composition of the nodule microbiome.

Although inoculation clearly enhanced plant performance, our data do not allow us to confirm that the applied strains dominated nodule occupancy. Table S3 shows that nodules from all species and treatments contained multiple bacterial taxa, including both the expected rhizobial symbionts and diverse non‑rhizobial endophytes. δ¹⁵N values were also low in non‑inoculated plants, indicating that indigenous rhizobia were active N fixers. Such patterns are typical in field soils where native rhizobia are abundant and competitive. Importantly, inoculation can still enhance plant performance without full nodule dominance, by increasing early colonization probability, altering competitive dynamics, or modifying rhizosphere processes. Definitive strain‑level attribution would require genomic markers or whole‑genome sequencing, which was beyond the scope of this field‑based study.

Soil microbial communities also shifted markedly with treatments. Light and inoculation promoted increases in *Bacteroidetes* and *Firmicutes*, phyla often associated with rapid nutrient turnover, whereas shaded and non-inoculated soils retained greater representation of *Acidobacteria*, which are typical of resource-limited environments [26, 29]. The high proportion of unclassified reads in shaded inoculated soils should be interpreted cautiously. Because our analysis is based on short‑read 16 S rRNA amplicons, unclassified sequences may reflect database gaps, limited phylogenetic resolution, or pipeline‑specific thresholds rather than the presence of truly novel microbial lineages. Therefore, while these patterns suggest shifts in community structure [30] associated to light and shade, they do not allow us to infer taxonomic novelty without complementary approaches such as full‑length 16 S sequencing or shotgun metagenomics.

The vegetation data demonstrate that inoculation and shade synergistically enhanced diversity and richness, particularly in the two annual species, *V. sativa* and *O. compressus* plots, a fact that had been proven before in other leguminous species in the Iberian Peninsula [9, 31–34]. These results suggest that conservative conditions (shade) buffered survival and coexistence, while inoculation enhanced resource availability and biomass, promoting facilitative interactions. In contrast, light-exposed plots generally supported lower richness, consistent with acquisitive strategies that intensify competition for resources. Together, these findings confirm that legume strategies mediated by inoculation and light filter the structure of plant communities, in agreement with PES predictions that resource acquisitiveness can reduce coexistence, while conservative strategies enhance it.

The contrasting ecological origins of the studied legumes help explain the species-specific responses to light, inoculation, and associated microbial dynamics. *T. repens* and *V. sativa*, both originating from shaded environments, showed higher survival and root allocation under shade, reflecting a conservative strategy typical of species adapted to resource-limited, low-light conditions [6]. Their greater responsiveness to inoculation under shade further highlights the importance of efficient symbiotic associations when carbon availability is constrained. In contrast, *O. compressus* and *C. juncea*, native to well-irradiated habitats, performed better under high light, with enhanced biomass, nodulation, and nitrogen accumulation, indicative of an acquisitive strategy optimized for rapid resource uptake in light-rich environments. Importantly, these ecological origins also influenced microbial diversity: shaded-origin legumes were associated with higher proportions of unclassified or novel microbial taxa in shaded inoculated plots, suggesting niche-specific recruitment under conservative growth strategies, while light-origin species supported more diverse and abundant microbial communities in illuminated, non-inoculated soils, reflecting their adaptation to high-resource turnover environments [7]. Together, these findings underscore that ecological origin shapes not only plant performance but also the structure of belowground microbial assemblages, reinforcing the linkage between conservative versus acquisitive strategies across the Plant Economic Spectrum.

Overall, our results demonstrate that inoculation acts as a biotic driver shifting plants toward acquisitive strategies, characterized by higher biomass, faster growth, greater nodulation, and enhanced N acquisition. In contrast, shade acts as an abiotic filter reinforcing conservative strategies, characterized by higher survival, root allocation, microbial diversity, and species coexistence. Species varied in their degree of plasticity, with annuals (*V. sativa*, *O. compressus*) showing stronger acquisitive responses to inoculation and light, and perennials (*T. repens*) showing more gradual, conservative adjustments. By contrast, *C. juncea* exhibited minimal plasticity and weak responses to both light and inoculation, maintaining a consistently conservative, stress‑tolerant strategy regardless of treatment. Thus, inoculation and light jointly determine the position of legumes along the PES continuum, with cascading effects on soil microbiomes and plant community assembly.

A limitation of our microbial analysis is that 16S rRNA sequencing was conducted only for soils associated with *T. repens* and only at the final sampling point (2024). Although we included a 2019 baseline and a 2024 control (Fig. 5b), which provide valuable temporal context, the dataset does not constitute a full temporal series nor a cross‑species comparison. For this reason, the microbial results should be interpreted as exploratory. Nevertheless, the observed shifts in phylum‑level composition between inoculated and non‑inoculated soils, and between light treatments, are consistent with known ecological responses to legume–rhizobia interactions and light‑driven changes in soil resource availability.”

## Supplementary Information

Below is the link to the electronic supplementary material.

Supplementary Material 1

## Data Availability

No datasets were generated or analysed during the current study.
