# Supplementary material for: The Interplay of Light and Microbial Symbiosis in Shaping Plant Economic Spectrum Strategies
Source: Microb Ecol. 2026 May 8;89(1):139. doi: 10.1007/s00248-026-02777-4 (PMC13323461; doi:10.1007/s00248-026-02777-4)
Supplement: Supplementary file 1 — Supplementary Material 1 [file 248_2026_2777_MOESM1_ESM.docx]

**Table S1.** Climatic data of the study site over the five-year period of study.

| **Month** | **Average temperature (ºC)** | **Minimum Temperature (ºC)** | **Maximum Temperature (ºC)** | | **Rainfall (mm)** | | **Relative Humidity (%)** | |
| --- | --- | --- | --- | --- | --- | --- | --- | --- |
| **2019** |  |  |  | |  | |  | |
| March  April  May  June  July  August  September  October  November  December | 13.60  13.40  17.00  22.50  26.60  27.20  25.00  19.80  15.20  11.610 | 6.80  6.10  8.70  12.50  15.90  17.20  14.30  10.40  6.80  3.40 | 20.60  22.70  25.60  33.00  36.90  37.30  34.30  29.50  23.70  19.40 | 105.40  80.20  36010  14.20  1.60  9.10  33.40  77.80  97.10  80.40 | | 78.90  77.50  67.90  53.40  43.20  48.80  73.10  84.60  86.90  85.880 | |  |
| **2020** |  |  |  |  | |  | |  |
| January  February  March  April  May  June  July  August  September  October  November  December | 11.46  12.1  12.95  15.22  20.61  22.58  29.22  26.15  23.36  16.54  13.75  9.83 | 1.80  19.50  0.10  1.50  9.70  8.80  12.60  11.40  9.60  4.10  4.40  -3.30 | 25.50  6.00  27.30  26.60  35.40  40.30  42.40  41.50  38.80  32.70  24.00  18.90 | 3.80  0.50  74.60  115.60  38.60  0  4.20  0  16.60  52.40  133.80  45.20 | | 83.36  77.90  76.86  80.69  68.79  56.46  43.78  49.59  54.05  66.80  87.85  89.23 | |  |
| 2021 |  |  |  |  | |  | |  |
| January  February  March  April  May  June  July  August  September  October  November  December | 6.98  11.89  12.95  16.13  18.83  22.51  25.35  26.8  22.27  18.36  10.09  10.81 | -5.20  3.70  0.30  5.80  4.50  7.70  12.00  11.80  9.40  4.80  0.30  -0.90 | 17.80  21.90  31.40  27.70  35.60  37.20  41.10  44.70  38.50  31.80  22.60  20.80 | 51.40  106.40  11.40  76.00  19.00  41.40  0  0.40  53.40  44.60  11.20  41.20 | | 87.72  84.40  70.19  76.26  61.51  57.58  51.17  50.19  64.21  67.64  78.79  86.38 | |  |
| **2022** |  |  |  |  | |  | |  |
| January  February  March  April  May  June  July  August  September  October  November  December | 7.70  10.4  12.41  13.80  22.23  24.13  29.57  27.11  22.71  20.14  13.58  12.58 | -2.60  -1.50  -0.10  2.00  8.80  11.00  13.10  13.40  9.90  9.20  1.50  1.60 | 21.00  23.80  22.50  30.80  37.40  41.60  45.20  42.40  36.80  34.10  23.10  20.00 | 0.80  1.00  35.80  29.40  2.00  13.60  0  0  45.80  72.60  63.40  247.60 | | 79.65  71.86  75.70  69.55  51.81  52.70  40.4  48.47  62.32  67.69  87.59  91.43 | |  |
| **2023** |  |  |  |  | |  | |  |
| January  February  March  April  May  June  July  August  September  October  November  December | 8.13  9.16  13.70  18.65  20.55  25.58  26.93  28.31  22.42  20.00  13.38  8.63 | -3.20  -2.90  -3.70  2.70  8.50  13.10  13.90  14.70  9.80  9.30  0.80  -1.00 | 20.80  21.40  29.30  35.30  34.90  42.30  41.30  44.70  37.90  38.00  23.20  20.4 | 34.00  9.20  20.20  4.80  37.20  5.40  0  0  25.00  177.6  51.00  18.40 | | 85.21  71.40  71.49  53.88  54.00  55.20  46.72  44.60  63.25  68.24  87.44  90.99 | |  |
| **2024** |  |  |  |  | |  | |  |
| January  February  March  April  May  June | 10.44  11.81  12.90  16.52  19.27  23.16 | -1.40  1.50  1.50  4.50  4.10  10.70 | 21.20  22.9  26.8  31.10  38.70  39.30 | 140.4  46.2  110.8  18.2  1.40  23.2 | | 90.29  84.35  81.16  70.92  59.75  59.67 | |  |

**Table S2**. Herbaceous species identified in plots planted with inoculated and non-inoculated seedlings in full light and in the shade. Plant inventories were conducted each year after planting. C: Control plot; NI: Non-inoculated seedlings; I: inoculated seedlings.

|  | **Control** |
| --- | --- |
| 2019 | *Avena sterilis* L.  *Bromus hordeaceus* L.  *Anacyclus clavatus* (Desf.) Pers.  *Chondrilla juncea* L. |
| 2021 | *Malva sylvestris* L.  *Taraxacum vulgare* (Lam.) Schrank  *Trifolium campestre* Schreb  *Melilotus indicus* (L.) All.  *Geranium robertianum* L.  *Hypochaeris radicata* L.  *Senecio vulgaris* L.  *Fumaria officinalis* L.  *Raphanus raphanistrum* L. |
| 2022 | *Avena sterilis* L.  *Malva sylvestris* L.  *Cynodon dactylon* (L.) Pers*.*  *Taraxacum vulgare* (Lam.) Schrank  *Senecio vulgaris* L*.*  *Bromus rubens* L.  *Hordeum murinum* L.  *Lolium perenne* L.  *Geranium molle* L.  *Euphorbia hirsuta* L.  *Trifolium campestre* Schreb |
| 2023 | *Avena sterilis* L.  *Malva sylvestris* L.  *Diplotaxis erucoides* (L.) DC. *Cynodon dactylon* (L.) Pers*.*  *Taraxacum vulgare* (Lam.) Schrank  *Senecio vulgaris* L*.*  *Bromus rubens* L.  *Hordeum murinum* L.  *Lolium perenne* L.  *Geranium molle* L.  *Trifolium tomentosum* L.  *Trifolium campestre* Schreb |
| 2024 | *Cynodon dactylon* (L.) Pers*.*  *Taraxacum vulgare* (Lam.) Schrank  *Geranium molle* L.  *Trifolium tomentosum* L.  *Trifolium campestre* Schreb  *Plantago lanceolata* L. |

|  | ***T. repens*** |  |  |  |
| --- | --- | --- | --- | --- |
|  | **Non Inoculated** |  | **Inoculated** |  |
|  | **Shade** | **Light** | **Shade** | **Light** |
| 2021 | *Geranium molle* L.  *Fumaria officinalis* L.  *Stellaria media* (L.) Vill.  *Capsella bursa-pastoris* (L.) Medik.  *Veronica persica* Poir. in Lam.  *Malva sylvestris* L.  *Anagallis arvensis* L.  *Mercurialis annua* L.  *Galium aparine* L.  *Plantago major* L.  *Silene vulgaris* (Moench) Garcke  *Papaver rhoeas* L.  *Trifolium campestre* Schreb  *Matricaria chamomilla* L.  *Chenopodium album* L. | *Raphanus raphanistrum* [L.](https://es.wikipedia.org/wiki/Carlos_Linneo)  *Fumaria parviflora* Lam.  *Sinapis arvensis* L.  *Veronica persica* Poir. in Lam.  *Malva sylvestris* L.  *Anagallis arvensis* L.  *Diplotaxis erucoides* (L.) DC.  *Galium aparine* L.  *Plantago lanceolata* L.  *Silene vulgaris* (Moench) Garcke  *Papaver rhoeas* L.  *Trifolium campestre* Schreb  *Matricaria chamomilla* L.  *Chenopodium album* L.  *Sonchus oleraceus* L.  *Amaranthus retroflexus* L.  *Convolvulus arvensis* L. | *Sinapis arvensis* L.  *Veronica persica* Poir. in Lam.  *Papaver rhoeas* L.  *Trifolium campestre* Schreb  *Capsella bursa-pastoris* (L.) Medik.  *Fumaria officinalis* L.  *Lolium rigidum* Gaudin  *Convolvulus arvensis* L.  *Chenopodium album* L.  *Diplotaxis erucoides* (L.) DC.  *Geranium rotundifolium* L.  *Matricaria chamomilla* L.  *Aegilops geniculata* Roth  *Malva sylvestris* L.  *Anagallis arvensis* L.  *Plantago major* L.  *Vicia sativa* L. | *Cynodon dactylon* (L.) Pers.  *Taraxacum vulgare* Schrank  *Geranium molle* L.  *Trifolium tomentosum* L.  *Trifolium campestre* Schreb  *Plantago lanceolata* L.  *Amaranthus retroflexus* L.  *Anagallis arvensis* L.  *Chenopodium album* L.  *Fumaria officinalis* L.  *Fumaria parviflora* Lam.  *Mercurialis annua* L.  *Capsella bursa-pastoris* (L.) Medik.  *Stellaria media* (L.) Vill.  *Veronica persica* Poir. in Lam.  *Polygonum aviculare* L.  *Sonchus oleraceus* L.  *Sinapis arvensis* L.  *Papaver rhoeas* L.  *Matricaria chamomilla* L.  *Galium aparine* L.  *Silene vulgaris* (Moench) Garcke  *Euphorbia helioscopia* L.  *Malva sylvestris* L.  *Convolvulus arvensis* L.  *Lolium rigidum* Gaudin  *Avena sterilis* L.  *Diplotaxis erucoides* (L.) DC.  *Scandix pecten-veneris* L.  *Medicago polymorpha* L.  *Vicia villosa* Roth  *Tribulus terrestris* L. |
| 2022 | *Geranium molle* L.  *Fumaria officinalis* L.  *Stellaria media* (L.) Vill.  *Capsella bursa-pastoris* (L.) Medik.  *Veronica persica* Poir. in Lam.  *Malva sylvestris* L.  *Anagallis arvensis* L.  *Mercurialis annua* L.  *Galium aparine* L.  *Plantago major* L.  *Silene vulgaris* (Moench) Garcke  *Papaver rhoeas* L.  *Scleranthus annuus* L. | *Geranium rotundifolium* L.  *Fumaria parviflora* Lam.  *Sinapis arvensis* L.  *Veronica persica* Poir. in Lam.  *Malva sylvestris* L.  *Anagallis arvensis* L.  *Diplotaxis erucoides* (L.) DC.  *Galium aparine* L.  *Plantago lanceolata* L.  *Papaver rhoeas* L.  *Chenopodium album* L.  *Sonchus oleraceus* L.  *Convolvulus arvensis* L.  *Matricaria chamomilla* L. | *Diplotaxis erucoides* (L.) DC.  *Papaver rhoeas* L.  *Veronica persica* Poir. in Lam.  *Trifolium campestre* Schreb  *Capsella bursa-pastoris* (L.) Medik.  *Fumaria officinalis* L.  *Lolium rigidum* Gaudin  *Convolvulus arvensis* L.  *Chenopodium album* L.  *Geranium rotundifolium* L.  *Matricaria chamomilla* L.  *Aegilops geniculata* Roth  *Malva sylvestris* L.  *Anagallis arvensis* L.  *Plantago major* L.  *Polygonum aviculare* L. | *Cynodon dactylon* (L.) Pers.  *Taraxacum vulgare* Schrank  *Geranium molle* L.  *Trifolium tomentosum* L.  *Trifolium campestre* Schreb  *Plantago lanceolata* L.  *Amaranthus retroflexus* L.  *Anagallis arvensis* L.  *Chenopodium album* L.  *Fumaria officinalis* L.  *Fumaria parviflora* Lam.  *Mercurialis annua* L.  *Capsella bursa-pastoris* (L.) Medik*.*  *Stellaria media* (L.) Vill.  *Veronica persica* Poir. in Lam.  *Polygonum aviculare* L.  *Sonchus oleraceus* L.  *Sinapis arvensis* L.  *Papaver rhoeas* L.  *Matricaria chamomilla* L.  *Galium aparine* L.  *Silene vulgaris* (Moench) Garcke  *Euphorbia helioscopia* L.  *Malva sylvestris* L.  *Convolvulus arvensis* L.  *Lolium rigidum* Gaudin  *Bromus rubens* L.  *Avena sterilis* L.  *Hordeum murinum* L.  *Diplotaxis erucoides* (L.) DC.  *Salsola kali* L.  *Scandix pecten-veneris* L.  *Lathyrus aphaca* L.  *Medicago polymorpha* L.  *Vicia villosa* Roth  *Vicia sativa* L.  *Trifolium subterraneum* L. |
| 2023 | *Geranium molle* L.  *Fumaria officinalis* L.  *Stellaria media* (L.) Vill.  *Capsella bursa-pastoris* (L.) Medik.  *Veronica persica* Poir. in Lam.  *Malva sylvestris* L.  *Anagallis arvensis* L.  *Mercurialis annua* L.  *Galium aparine* L.  *Plantago major* L.  *Silene vulgaris* (Moench) Garcke  *Papaver rhoeas* L.  *Scleranthus annuus* L.  *Trifolium campestre* Schreb | *Geranium rotundifolium* L.  *Fumaria parviflora* Lam.  *Sinapis arvensis* L.  *Veronica persica* Poir. in Lam.  *Malva sylvestris* L.  *Anagallis arvensis* L.  *Diplotaxis erucoides* (L.) DC.  *Galium aparine* L.  *Plantago lanceolata* L.  *Papaver rhoeas* L.  *Trifolium campestre* Schreb  *Matricaria chamomilla* L.  *Chenopodium album* L.  *Sonchus oleraceus* L.  *Convolvulus arvensis* L. | *Stellaria media* (L.) Vill  *Capsella bursa-pastoris* (L.) Medik.  *Veronica persica* Poir. in Lam.  *Galium aparine* L.  *Fumaria officinalis* L.  *Malva sylvestris* L.  *Anagallis arvensis* L.  *Mercurialis annua* L.  *Plantago major* L.  *Silene vulgaris* (Moench) Garcke  *Papaver rhoeas* L.  *Trifolium campestre* Schreb | *Sinapis arvensis* L.  *Diplotaxis erucoides* (L.) DC.  *Chenopodium album* L.  *Sonchus oleraceus* L.  *Veronica persica* Poir. in Lam.  *Galium aparine* L.  *Fumaria parviflora* Lam.  *Malva sylvestris* L.  *Anagallis arvensis* L.  *Plantago lanceolata* L.  *Papaver rhoeas* L.  *Trifolium campestre* Schreb  *Matricaria chamomilla* L.  *Convolvulus arvensis* L.  *Amaranthus retroflexus* L.  *Lolium rigidum* Gaudin  *Avena sterilis* L.  *Geranium rotundifolium* L.  *Euphorbia helioscopia* L.  *Capsella bursa-pastoris* (L.) Medik. |
| 2024 | *Cynodon dactylon* (L.) Pers*.*  *Taraxacum vulgare* (Lam.) Schrank  *Geranium molle* L.  *Trifolium tomentosum* L.  *Trifolium campestre* Schreb  *Plantago lanceolata* L.  *Senecio vulgaris* L.  *Convolvulus arvensis* L.  *Anagallis arvensis* L.  *Chenopodium album* L.  *Fumaria officinalis* L.  *Polygonum aviculare* L. | *Cynodon dactylon* (L.) Pers*.*  *Taraxacum vulgare* (Lam.) Schrank  *Geranium molle* L.  *Trifolium tomentosum* L.  *Trifolium campestre* Schreb  *Plantago lanceolata* L.  *Amaranthus retroflexus* L. *Anagallis arvensis* L.  *Chenopodium album* L.  *Fumaria officinalis* L. *Fumaria parviflora* Lam.  *Mercurialis annua* L. | *Cynodon dactylon* (L.) Pers*.*  *Taraxacum vulgare* (Lam.) Schrank  *Geranium molle* L.  *Trifolium tomentosum* L.  *Trifolium campestre* Schreb  *Plantago lanceolata* L.  *Senecio vulgaris* L.  *Convolvulus arvensis* L.  *Anagallis arvensis* L.  *Chenopodium album* L.  *Fumaria officinalis* L.  *Asparagus officinalis* L. | *Arenaria montana* L.  *Paronychia argentea* Lam.  *Cynodon dactylon* (L.) Pers*.*  *Taraxacum vulgare* (Lam.) Schrank  *Geranium molle* L.  *Trifolium tomentosum* L.  *Trifolium campestre* Schreb  *Plantago lanceolata* L.  *Picris echioides* L.  *Scolymus hispanicus* L. |

|  | ***V. sativa*** |  |  |  | |  |
| --- | --- | --- | --- | --- | --- | --- |
|  | **Non Inoculated** |  | **Inoculated** |  | |  |
|  | **Shade** | **Light** | **Shade** | **Light** | |  |
| 2021 | *Sinapis arvensis* L.  *Diplotaxis erucoides* (L.) DC.  *Chenopodium album* L.  *Sonchus oleraceus* L.  *Veronica persica* Poir. in Lam.  *Galium aparine* L.  *Fumaria parviflora* Lam.  *Malva sylvestris* L.  *Anagallis arvensis* L.  *Plantago lanceolata* L.  *Papaver rhoeas* L.  *Trifolium campestre* Schreb  *Matricaria chamomilla* L.  *Convolvulus arvensis* L.  *Amaranthus retroflexus* L.  *Lolium rigidum* Gaudin  *Avena sterilis* L.  *Geranium rotundifolium* L.  *Euphorbia helioscopia* L.  *Capsella bursa-pastoris* (L.) Medik. | *Geranium rotundifolium* L.  *Fumaria parviflora* Lam.  *Sinapis arvensis* L.  *Diplotaxis erucoides* (L.) DC.  *Trifolium campestre* Schreb  *Papaver rhoeas* L.  *Plantago lanceolata* L.  *Anagallis arvensis* L.  *Convolvulus arvensis* L.  *Matricaria chamomilla* L. | *Stellaria media* (L.) Vill.  *Capsella bursa-pastoris* (L.) Medik.  *Veronica persica* Poir. in Lam.  *Galium aparine* L.  *Fumaria officinalis* L.  *Malva sylvestris* L.  *Anagallis arvensis* L.  *Mercurialis annua* L.  *Plantago major* L.  *Silene vulgaris* (Moench) Garcke  *Papaver rhoeas* L.  *Trifolium campestre* Schreb  *Chenopodium album* L. | *Sinapis arvensis* L.  *Diplotaxis erucoides* (L.) DC.  *Chenopodium album* L.  *Sonchus oleraceus* L.  *Veronica persica* Poir. in Lam.  *Galium aparine* L.  *Fumaria parviflora* Lam.  *Malva sylvestris* L.  *Anagallis arvensis* L.  *Plantago lanceolata* L.  *Papaver rhoeas* L.  *Trifolium campestre* Schreb  *Matricaria chamomilla* L.  *Convolvulus arvensis* L.  *Amaranthus retroflexus* L.  *Lolium rigidum* Gaudin  *Avena sterilis* L.  *Geranium rotundifolium* L.  *Euphorbia helioscopia* L.  *Capsella bursa-pastoris* (L.) Medik.  *Fumaria officinalis* L.  *Silene vulgaris* (Moench) Garcke | |  |
| 2022 | *Geranium molle* L.  *Fumaria officinalis* L.  *Capsella bursa-pastoris* (L.) Medik.  *Veronica persica* Poir. in Lam.  *Scleranthus annuus* L.  *Plantago major* L.  *Silene vulgaris* (Moench) Garcke  *Trifolium campestre* Schreb  *Mercurialis annua* L. | *Geranium rotundifolium* L.  *Fumaria parviflora* Lam.  *Sinapis arvensis* L.  *Diplotaxis erucoides* (L.) DC.  *Trifolium campestre* Schreb  *Papaver rhoeas* L.  *Plantago lanceolata* L.  *Anagallis arvensis* L.  *Convolvulus arvensis* L.  *Matricaria chamomilla* L.  *Silene vulgaris* (Moench) Garcke  *Avena sterilis* L.  *Raphanus raphanistrum* L. | *Stellaria media* (L.) Vill.  *Capsella bursa-pastoris* (L.) Medik.  *Veronica persica* Poir. in Lam.  *Galium aparine* L.  *Fumaria officinalis* L.  *Malva sylvestris* L.  *Anagallis arvensis* L.  *Mercurialis annua* L.  *Plantago major* L.  *Silene vulgaris* (Moench) Garcke  *Papaver rhoeas* L.  *Trifolium campestre* Schreb  *Chenopodium album* L.  *Matricaria chamomilla* L.  *Sonchus oleraceus* L.  *Geranium molle* L.  *Euphorbia helioscopia* L.  *Veronica hederifolia* L.  *Plantago coronopus* L. | *Sinapis arvensis* L.  *Diplotaxis erucoides* (L.) DC.  *Chenopodium album* L.  *Sonchus oleraceus* L.  *Veronica persica* Poir. in Lam.  *Galium aparine* L.  *Fumaria parviflora* Lam.  *Malva sylvestris* L.  *Anagallis arvensis* L.  *Plantago lanceolata* L.  *Papaver rhoeas* L.  *Trifolium campestre* Schreb  *Matricaria chamomilla* L.  *Convolvulus arvensis* L.  *Amaranthus retroflexus* L.  *Lolium rigidum* Gaudin  *Avena sterilis* L.  *Geranium rotundifolium* L.  *Euphorbia helioscopia* L.  *Capsella bursa-pastoris* (L.) Medik.  *Fumaria officinalis* L.  *Silene vulgaris* (Moench) Garcke  *Kickxia spuria* (L.) Dumort.  *Polygonum aviculare* L.  *Medicago polymorpha* L.  *Veronica hederifolia* L. | |  |
| 2023 | *Geranium molle* L.  *Fumaria officinalis* L.  *Capsella bursa-pastoris* (L.) Medik.  *Veronica persica* Poir. in Lam.  *Scleranthus annuus* L.  *Plantago major* L.  *Silene vulgaris* (Moench) Garcke  *Trifolium campestre* Schreb  *Mercurialis annua* L.  *Anagallis arvensis* L.  *Galium aparine* L.  *Papaver rhoeas* L.  *Matricaria chamomilla* L.  *Stellaria media* (L.) Vill.  *Veronica hederifolia* L.  *Plantago coronopus* L.  *Geranium pusillum* L.  *Medicago polymorpha* L. | *Geranium rotundifolium* L.  *Fumaria parviflora* Lam.  *Sinapis arvensis* L.  *Diplotaxis erucoides* (L.) DC.  *Trifolium campestre* Schreb  *Papaver rhoeas* L.  *Plantago lanceolata* L.  *Anagallis arvensis* L.  *Convolvulus arvensis* L.  *Matricaria chamomilla* L.  *Silene vulgaris* (Moench) Garcke  *Avena sterilis* L.  *Kickxia elatine* (L.) Dumort.  *Polygonum aviculare* L.  *Euphorbia helioscopia* L.  *Lolium rigidum* Gaudin  *Vicia sativa* L.  *Medicago polymorpha* L.  *Malva sylvestris* L. | *Stellaria media* (L.) Vill.  *Capsella bursa-pastoris* (L.) Medik.  *Veronica persica* Poir. in Lam.  *Galium aparine* L.  *Chenopodium album* L.  *Sonchus oleraceus* L.  *Fumaria officinalis* L.  *Malva sylvestris* L.  *Mercurialis annua* L.  *Papaver rhoeas* L.  *Plantago major* L. | *Sinapis arvensis* L.  *Diplotaxis erucoides* (L.) DC.  *Chenopodium album* L.  *Sonchus oleraceus* L.  *Veronica persica* Poir. in Lam.  *Galium aparine* L.  *Fumaria parviflora* Lam.  *Malva sylvestris* L.  *Anagallis arvensis* L.  *Plantago lanceolata* L.  *Papaver rhoeas* L.  *Trifolium campestre* Schreb  *Matricaria chamomilla* L.  *Convolvulus arvensis* L.  *Amaranthus retroflexus* L.  *Lolium rigidum* Gaudin  *Avena sterilis* L.  *Geranium rotundifolium* L.  *Euphorbia helioscopia* L.  *Capsella bursa-pastoris* (L.) Medik.  *Fumaria officinalis* L.  *Silene vulgaris* (Moench) Garcke  *Raphanus raphanistrum* L.  *Polygonum aviculare* L.  *Medicago polymorpha* L.  *Veronica hederifolia* L. | |  |
| 2024 | *Geranium molle* L.  *Fumaria officinalis* L.  *Capsella bursa-pastoris* (L.) Medik.  *Veronica persica* Poir. in Lam.  *Scleranthus annuus* L.  *Plantago major* L.  *Silene vulgaris* (Moench) Garcke  *Trifolium campestre* Schreb  *Mercurialis annua* L.  *Anagallis arvensis* L.  *Galium aparine* L.  *Papaver rhoeas* L.  *Matricaria chamomilla* L.  *Raphanus raphanistrum* L. | *Geranium rotundifolium* L.  *Fumaria parviflora* Lam.  *Sinapis arvensis* L.  *Diplotaxis erucoides* (L.) DC.  *Trifolium campestre* Schreb  *Papaver rhoeas* L.  *Plantago lanceolata* L.  *Anagallis arvensis* L.  *Convolvulus arvensis* L.  *Matricaria chamomilla* L.  *Silene vulgaris* (Moench) Garcke  *Avena sterilis* L.  *Kickxia elatine* (L.) Dumort.  *Polygonum aviculare* L.  *Euphorbia helioscopia* L.  *Malva sylvestris* L. | *Stellaria media* (L.) Vill.  *Capsella bursa-pastoris* (L.) Medik.  *Veronica persica* Poir. in Lam.  *Galium aparine* L.  *Chenopodium album* L.  *Sonchus oleraceus* L.  *Fumaria officinalis* L.  *Malva sylvestris* L.  *Plantago major* L. | *Sinapis arvensis* L.  *Diplotaxis erucoides* (L.) DC.  *Chenopodium album* L.  *Sonchus oleraceus* L.  *Veronica persica* Poir. in Lam.  *Galium aparine* L.  *Fumaria parviflora* Lam.  *Malva sylvestris* L.  *Anagallis arvensis* L.  *Plantago lanceolata* L.  *Papaver rhoeas* L.  *Trifolium campestre* Schreb  *Matricaria chamomilla* L.  *Convolvulus arvensis* L.  *Amaranthus retroflexus* L.  *Capsella bursa-pastoris* (L.) Medik.  *Euphorbia helioscopia* L. | |  |
|  | ***O. compressus*** |  |  |  | | |
|  | **Non Inoculated** |  | **Inoculated** |  | | |
|  | **Shade** | **Light** | **Shade** | **Light** | | |
| 2021 | *Geranium molle* L.  *Scleranthus annuus* L.  *Plantago major* L. | *Geranium rotundifolium* L.  *Fumaria parviflora* Lam.  *Sinapis arvensis* L.  *Diplotaxis erucoides* (L.) DC.  *Trifolium campestre* Schreb  *Papaver rhoeas* L.  *Plantago lanceolata* L.  *Anagallis arvensis* L.  *Convolvulus arvensis* L.  *Matricaria chamomilla* L.  *Raphanus raphanistrum* L. | *Stellaria media* (L.) Vill.  *Capsella bursa-pastoris* (L.) Medik.  *Veronica persica* Poir. in Lam.  *Galium aparine* L.  *Chenopodium album* L.  *Sonchus oleraceus* L.  *Fumaria officinalis* L.  *Malva sylvestris* L.  *Mercurialis annua* L.  *Papaver rhoeas* L.  *Plantago major* L. | *Sinapis arvensis* L.  *Diplotaxis erucoides* (L.) DC.  *Chenopodium album* L.  *Sonchus oleraceus* L.  *Veronica persica* Poir. in Lam.  *Galium aparine* L.  *Fumaria parviflora* Lam.  *Malva sylvestris* L.  *Anagallis arvensis* L.  *Plantago lanceolata* L.  *Papaver rhoeas* L.  *Trifolium campestre* Schreb  *Matricaria chamomilla* L.  *Convolvulus arvensis* L.  *Amaranthus retroflexus* L.  *Capsella bursa-pastoris* (L.) Medik. | | |
| 2022 | *Geranium molle* L.  *Fumaria officinalis* L.  *Capsella bursa-pastoris* (L.) Medik.  *Veronica persica* Poir. in Lam.  *Scleranthus annuus* L.  *Plantago major* L.  *Silene vulgaris* (Moench) Garcke  *Trifolium campestre* Schreb  *Mercurialis annua* L.  *Anagallis arvensis* L.  *Galium aparine* L.  *Papaver rhoeas* L.  *Matricaria chamomilla* L.  *Raphanus raphanistrum* | *Geranium rotundifolium* L.  *Fumaria parviflora* Lam.  *Sinapis arvensis* L.  *Diplotaxis erucoides* (L.) DC.  *Trifolium campestre* Schreb  *Papaver rhoeas* L.  *Plantago lanceolata* L.  *Anagallis arvensis* L.  *Convolvulus arvensis* L.  *Matricaria chamomilla* L.  *Silene vulgaris* (Moench) Garcke  *Avena sterilis* L.  *Kickxia elatine* (L.) Dumort.  *Polygonum aviculare* L.  *Euphobia helioscopia* L.  *Lolium rigidum* Gaudin  *Vicia sativa* L.  *Medicago polymorpha* L.  *Malva sylvestris* L. | *Geranium rotundifolium* L.  *Fumaria parviflora* Lam.  *Sinapis arvensis* L.  *Diplotaxis erucoides* (L.) DC.  *Trifolium campestre* Schreb  *Papaver rhoeas* L.  *Plantago lanceolata* L.  *Anagallis arvensis* L.  *Convolvulus arvensis* L.  *Matricaria chamomilla* L.  *Silene vulgaris* (Moench) Garcke  *Avena sterilis* L.  *Raphanus raphanistrum* L.  *Polygonum aviculare* L.  *Euphorbia helioscopia* L.  *Lolium rigidum* Gaudin  *Vicia sativa* L.  *Medicago polymorpha* L.  *Malva sylvestris* L. | *Sinapis arvensis* L.  *Diplotaxis erucoides* (L.) DC.  *Chenopodium album* L.  *Sonchus oleraceus* L.  *Veronica persica* Poir. in Lam.  *Galium aparine* L.  *Fumaria parviflora* Lam.  *Malva sylvestris* L.  *Anagallis arvensis* L.  *Plantago lanceolata* L.  *Papaver rhoeas* L.  *Trifolium campestre* Schreb  *Matricaria chamomilla* L.  *Convolvulus arvensis* L.  *Amaranthus retroflexus* L.  *Lolium rigidum* Gaudin  *Avena sterilis* L.  *Geranium rotundifolium* L.  *Euphorbia helioscopia* L.  *Capsella bursa-pastoris* (L.) Medik.  *Fumaria officinalis* L.  *Silene vulgaris* (Moench) Garcke  *Raphanus raphanistrum* L.  *Polygonum aviculare* L.  *Medicago polymorpha* L.  *Veronica hederifolia* L.  *Stellaria media* (L.) Vill.  *Vicia villosa* Roth  *Plantago coronopus* L. | | |
| 2023 | *Capsella bursa-pastoris (*L.) Medik.  *Stellaria media* (L.) Vill.  *Veronica persica* Poir. in Lam.  *Chenopodium album* L.  *Lamium amplexicaule* L.  *Sonchus oleraceus* L.  *Galium aparine* L.  *Plantago major* L.  *Polygonum aviculare* L.  *Fumaria officinalis* L.  *Anagallis arvensis* L.  *Papaver rhoeas* L.  *Matricaria chamomilla* L.  *Silene gallica* L | *Capsella bursa-pastoris* (L.) *Medik.*  *Stellaria media* (L.) Vill.  *Veronica persica* Poir. in Lam.  *Chenopodium album* L.  *Lamium amplexicaule* L*.*  *Sonchus oleraceus* L.  *Galium aparine* L.  *Plantago major* L.  *Polygonum aviculare L.*  *Fumaria officinalis* L.  *Anagallis arvensis* L.  *Papaver rhoeas* L.  *Matricaria chamomilla* L.  *Silene gallica* L.  *Amaranthus retroflexus* L.  *Mercurialis annua* L.  *Euphorbia peplus* L.  *Veronica hederifolia* L.  *Avena fatua* L.  *Polygonum lapathifolium* L.  *Malva sylvestris* L.  *Sisymbrium officinale* (L.) Scop- | *Capsella bursa-pastoris* (L.) *Medik. Stellaria media* (L.) Vill.  *Veronica persica* Poir. in Lam.  *Chenopodium album* L.  *Lamium amplexicaule* L.  *Sonchus oleraceus* L*.*  *Galium aparine* L.  *Plantago major* L*.*  *Polygonum aviculare* L.  *Fumaria officinalis* L.  *Anagallis arvensis* L.  *Papaver rhoeas* L.  *Matricaria chamomilla* L.  *Silene gallica* L.  *Amaranthus retroflexus* L.  *Mercurialis annua* L.  *Veronica hederifolia* L.  *Malva sylvestris* L.  *Sisymbrium officinale* (L.) Scop*.* | *Capsella bursa-pastoris* (L.) Medik.  *Stellaria media* (L.) Vill*.*  *Veronica persica* Poir. in Lam.  *Chenopodium album* L*.*  *Lamium amplexicaule* L.  *Sonchus oleraceus* L.  *Galium aparine* L.  *Plantago major* L.  *Polygonum aviculare* L.  *Fumaria officinalis* L*.*  *Anagallis arvensis* L.  *Papaver rhoeas* L*.*  *Matricaria chamomilla* L*.*  *Silene gallica* L.  *Amaranthus retroflexus* L.  *Mercurialis annua* L.  *Veronica hederifolia* L.  *Malva sylvestris* L.  *Sisymbrium officinale* (L.) Scop*.*  *Capsella bursa-pastoris (*L.) Medik.  *Stellaria media* (L.) Vill.  *Veronica persica* Poir. in Lam.  *Chenopodium album* L.  *Lamium amplexicaule* L.  *Sonchus oleraceus* L.  *Galium aparine* L. | | |
| 2024 | *Veronica persica* Poir. in Lam.  *Stellaria media* (L.) Vill.  *Capsella bursa-pastoris* (L.) Medik.  *Chenopodium album* L.  *Lamium amplexicaule* L.  *Sonchus oleraceus* L.  *Galium aparine* L.  *Plantago major* L.  *Papaver rhoeas* L.  *Mercurialis annua* L.  *Silene gallica* L. | *Veronica persica* Poir. in Lam.  *Stellaria media* (L.) Vill.  *Capsella bursa-pastoris* (L.) Medik.  *Chenopodium album* L.  *Lamium amplexicaule* L.  *Sonchus oleraceus*  *Galium aparine* L.  *Plantago major* L.  *Papaver rhoeas* L.  *Mercurialis annua* L.  *Silene gallica* L.  *Amaranthus retroflexus* L.  *Polygonum aviculare* L.  *Sinapis arvensis* L.  *Anagallis arvensis* L.  *Euphorbia peplus* L. | *Veronica persica* Poir. in Lam.  *Stellaria media* (L.) Vill.  *Capsella bursa-pastoris* (L.) Medik.  *Chenopodium album* L.  *Lamium amplexicaule* L.  *Sonchus oleraceus* L.  *Galium aparine* L.  *Plantago major* L.  *Papaver rhoeas* L.  *Mercurialis annua* L.  *Silene gallica* L.  *Amaranthus retroflexus* L.  *Polygonum aviculare* L.  *Sinapis arvensis* L.  *Anagallis arvensis* L.  *Euphorbia peplus* l.  *Veronica hederifolia* L.  *Polygonum lapathifolium* L. | *Veronica persica* Poir. in Lam.  *Stellaria media* (L.) Vill.  *Capsella bursa-pastoris* (L.) Medik.  *Chenopodium album* L.  *Lamium amplexicaule* L.  *Sonchus oleraceus* L.  *Galium aparine* L.  *Plantago major* L.  *Papaver rhoeas* L.  *Mercurialis annua* L.  *Silene gallica* L.  *Amaranthus retroflexus* L.  *Cynodon dactylon* (L.) Pers.  *Mercurialis annua* L.  *Veronica hederifolia* L.  *Malva sylvestris L.*  *Stellaria media* (L.) Vill.  *Lolium rigidum* Gaudin  *Sisymbrium officinale* (L.) Scop.  *Briza maxima* L.  *Plantago major* L. | | |
|  | ***C. juncea*** |  |  |  |  |  |
|  | **Non Inoculated** |  | **Inoculated** |  |  |  |
|  | **Shade** | **Light** | **Shade** | **Light** |  |  |
| 2021 | *Galium aparine* L.  *Polygonum aviculare* L.  *Capsella bursa-pastoris* (L.) Medik.  *Fumaria officinalis* L.  *Malva sylvestris* L. | *Galium aparine* L.  *Polygonum aviculare* L.  *Capsella bursa-pastoris* (L.) Medik.  *Fumaria officinalis* L.  *Malva sylvestris* L.  *Lamium amplexicaule* L.  *Veronica persica* Poir. in Lam.  *Sisymbrium officinale* (L.) Scop.  *Plantago major* L.  *Amaranthus retroflexus* L. | *Avena fatua* L.  *Polygonum lapathifolium* L.  *Vicia sativa* L.  *Datura stramonium* L.  *Lolium multiflorum* Lam.  *Raphanus raphanistrum* L.  *Lactuca serriola* L.  *Matricaria chamomilla* L.  *Anagallis arvensis* L.  *Silene gallica* L.  *Cerastium glomeratum* Thuill.  *Veronica hederifolia* L.  *Euphorbia peplus* L.  *Spergula arvensis* L. | *Hirschfeldia incana* (L.) Lagr.-Foss.  *Cynodon dactylon* (L.) Pers.  *Urtica dioica* L.  *Urtica urens* L.  *Atriplex patula* L.  *Kickxia spuria* (L.) Dumort.  *Saponaria vaccaria* L.  *Bromus diandrus* Roth  *Trifolium repens* L.  *Ornithopus compressus* L.  *Sinapis arvensis* L.  *Convolvulus arvensis* L.  *Portulaca oleracea* L.  *Oxalis pes-caprae* L.  *Hordeum murinum* L.  *Parietaria judaica* L.  *Coronopus didymus* (L.) Sm.  *Geranium dissectum* L.  *Fallopia convolvulus (*L.) Á. Löve  *Diplotaxis muralis* (L.) DC.  *Coronilla juncea* L. |  |  |
| 2022 | *Capsella bursa-pastoris* (L.) Medik.  *Stellaria media* (L.) Vill.  *Veronica persica* Poir. in Lam.  *Chenopodium album* L.  *Lamium amplexicaule* L.  *Sonchus oleraceus* L.  *Galium aparine* L.  *Plantago major* L.  *Polygonum aviculare* L.  *Fumaria officinalis* L.  *Anagallis arvensis* L.  *Papaver rhoeas* L.  *Matricaria chamomilla* L.  *Silene gallica* L. | *Capsella bursa-pastoris* (L.) Medik.  *Stellaria media* (L.) Vill.  *Veronica persica* Poir. in Lam.  *Chenopodium album* L.  *Lamium amplexicaule* L.  *Sonchus oleraceus* L.  *Galium aparine* L.  *Plantago major* L.  *Polygonum aviculare* L.  *Fumaria officinalis* L.  *Anagallis arvensis* L.  *Papaver rhoeas* L.  *Matricaria chamomilla* L.  *Silene gallica* L.  *Amaranthus retroflexus* L. *Mercurialis annua* L. | *Capsella bursa-pastoris* (L.) Medik.  *Stellaria media* (L.) Vill.  *Veronica persica* Poir. in Lam.  *Chenopodium album* L.  *Lamium amplexicaule* L.  *Sonchus oleraceus* L.  *Galium aparine* L.  *Plantago major* L.  *Polygonum aviculare* L.  *Fumaria officinalis* L.  *Anagallis arvensis* L.  *Papaver rhoeas* L.  *Matricaria chamomilla* L.  *Silene gallica* L.  *Amaranthus retroflexus* L.  *Mercurialis annua* L.  *Euphorbia peplus* L.  *Veronica hederifolia* L.  *Avena fatua* L. | *Capsella bursa-pastoris* (L.) Medik.  *Stellaria media* (L.) Vill.  *Veronica persica* Poir. in Lam.  *Chenopodium album* L.  *Lamium amplexicaule* L.  *Sonchus oleraceus* L.  *Galium aparine* L.  *Plantago major* L.  *Polygonum aviculare* L.  *Fumaria officinalis* L.  *Anagallis arvensis* L.  Papaver rhoeas L.  *Matricaria chamomilla* L.  *Silene gallica* L.  *Amaranthus retroflexus* L. *Mercurialis annua* L.  *Euphorbia peplus* L.  *Veronica hederifolia* L.  *Avena fatua* L.  *Polygonum lapathifolium* L.  *Malva sylvestris* L.  *Sisymbrium officinale* (L.) Scop.  *Spergula arvensis* L.  *Cerastium glomeratum* Thuill.  *Vicia sativa* L.  *Trifolium repens* L. |  |  |
| 2023 | *Vicia hirsuta* (L.) Gray  *Sinapis arvensis* L.  *Lolium rigidum* Gaudin  *Centaurea melitensis* L.  *Papaver dubium* L.  *Tragopogon dubius* Scop.  *Trifolium campestre* Schreb.  *Plantago lanceolata* L.  *Silene vulgaris* (Moench) Garcke  *Rumex crispus* L.  *Avena sterilis* L.  *Galium tricornutum* Dandy  *Sherardia arvensis* L.  *Anacyclus clavatus* (Desf.) Pers.  *Picris echioides* L.  *Torilis arvensis* (Huds.) Link | *Hirschfeldia incana* (L.) Lagr.-Foss.  *Amaranthus blitoides* S.Watson  *Mercurialis annua* L.  *Euphorbia peplus* L.  *Veronica hederifolia* L.  *Avena fatua* L.  *Polygonum lapathifolium* L.  *Malva sylvestris* L.  *Sisymbrium officinale* (L.) Scop.  *Spergula arvensis* L.  *Cerastium glomeratum* Thuill.  *Cynodon dactylon* (L.) Pers.  *Urtica dioica* L.  *Urtica urens* L.  *Fumaria officinalis* L.  *Anthemis arvensis* L.  *Bromus hordeaceus* L.  *Geranium rotundifolium* L.  *Melilotus albus* Medik.  *Conyza canadensis* (L.) Cronq.  *Daucus carota* L.  *Medicago polymorpha* L.  *Orobanche minor* Sm.  *Sonchus asper* (L.) Hill | *Stellaria media* (L.) Vill.  *Capsella bursa-pastoris* (L.) Medik.  *Veronica persica* Poir. in Lam.  *Chenopodium album* L.  *Lamium amplexicaule* L.  *Sonchus oleraceus* L.  *Galium aparine* L.  *Plantago major* L.  *Polygonum aviculare* L.  *Papaver rhoeas* L.  *Matricaria chamomilla* L.  *Silene gallica* L.  *Anagallis arvensis* L.  *Diplotaxis virgata* (Cav.) DC.  *Calendula arvensis* L.  *Kickxia spuria* (L.) Dumort. | *Vicia sativa* L.  *Trifolium repens* L.  *Ornithopus compressus* L.  *Coronilla juncea* L.  *Barlia robertiana* (Loisel.) Greuter  *Asphodelus fistulosus* L.  *Reseda luteola* L.  *Papaver hybridum* L.  *Picris hieracioides* L.  *Reichardia picroides* (L.) Roth  *Hordeum murinum* L.  *Bromus diandrus* Roth  *Lolium multiflorum* Lam.  *Phalaris minor* Retz.  *Lupinus albus* L.  *Tetragonia tetragonoides* (Pall.) Kuntze  *Silybum marianum* (L.) Gaertn.  *Cirsium arvense* (L.) Scop.  *Erodium cicutarium* (L.) L'Hér.  *Linaria vulgaris* Mill.  *Spergularia rubra* (L.) J.Presl & C.Presl  *Torilis nodosa* (L.) Gaertn.  *Ranunculus arvensis* L.  *Vaccaria hispanica* (Mill.) Rauschert  *Fallopia convolvulus* (L.) Á. Löve  *Thlaspi arvense* L.  *Rumex pulcher* L. |  |  |
| 2024 | *Vicia hirsuta* (L.) Gray  *Stellaria media* (L.) Vill.  *Capsella bursa-pastoris* (L.) Medik.  *Chenopodium album* L.  *Lamium amplexicaule* L.  *Galium aparine* L.  *Plantago major* L.  *Papaver rhoeas* L.  *Anagallis arvensis* L.  *Silene gallica* L. | *Hirschfeldia incana* (L.) Lagr.-Foss.  *Amaranthus retroflexus* L.  *Mercurialis annua* L.  *Euphorbia peplus* L.  *Veronica hederifolia* L.  *Avena fatua* L.  *Polygonum lapathifolium* L.  *Malva sylvestris* L.  *Sisymbrium officinale* (L.) Scop.  *Spergula arvensis* L.  *Cerastium glomeratum* Thuill.  *Cynodon dactylon* (L.) Pers.  *Urtica dioica* L.  *Fumaria officinalis* L.  *Polygonum aviculare* L.  *Matricaria chamomilla* L. | *Veronica persica* Poir. in Lam.  *Sonchus oleraceus* L.  *Sinapis arvensis* L.  *Lolium rigidum* Gaudin  *Centaurea melitensis* L.  *Tragopogon dubius* Scop.  *Trifolium campestre* Schreb.  *Plantago lanceolata* L.  *Rumex crispus* L.  *Avena sterilis* L.  *Picris echioides* L.  *Torilis arvensis* (Huds.) Link | *Vicia sativa* L.  *Trifolium repens* L.  *Ornithopus compressus* L.  *Coronilla juncea* L.  *Asphodelus fistulosus* L.  *Reseda luteola* L.  *Papaver dubium* L.  *Reichardia picroides* (L.) Roth  *Hordeum murinum* L.  *Bromus diandrus* Roth  *Lolium multiflorum* Lam.  *Phalaris minor* Retz.  *Silybum marianum* (L.) Gaertn.  *Cirsium arvense* (L.) Scop.  *Erodium cicutarium* (L.) L'Hér.  *Linaria vulgaris* Mill.  *Ranunculus arvensis* L.  *Fallopia convolvulus* (L.) Á. Löve  *Rumex pulcher L.* |  |  |

**Table S3**. Bacterial strains isolated from nodules of *T. repens*, *V. sativa*, *O. compressus* and *C. juncea* grown in the field, inoculated and non-inoculated both in full light and in the shade NI S: non-inoculated in the shade; IS: inoculated in the shade; NI L: non-inoculated in the light and; I L: inoculated in the light).

| **Plant species** | **Rhizobial strains** | **Other bacteria** | |
| --- | --- | --- | --- |
| *T. repens* | *Rhizobium leguminosarum* bv. *trifolii* | *Bacillus subtilis*, *Pseudomonas putida*, *Paenibacillus*, |  |
| *V. sativa* | *Rhizobium leguminosarum* bv. *viciae* | *Pseudomonas putida*, *Bacillus cereus*, *Serratia*, *Stenotrophomonas* spp. |  |
| *O. compressus* | *Mesorhizobium loti*, *Mesorhizobium mediterraneum* | *Bacillus thuringiensis*, *Pseudomonas spp*, *Microbacterium* spp.  *Enterobacter* spp. |  |
| *C. juncea* | *Bradyrhizobium* spp., *Rhizobium* spp. | *Bacillus megaterium*, *Pseudomonas aeruginosa*, *Azospirillum* spp. |  |


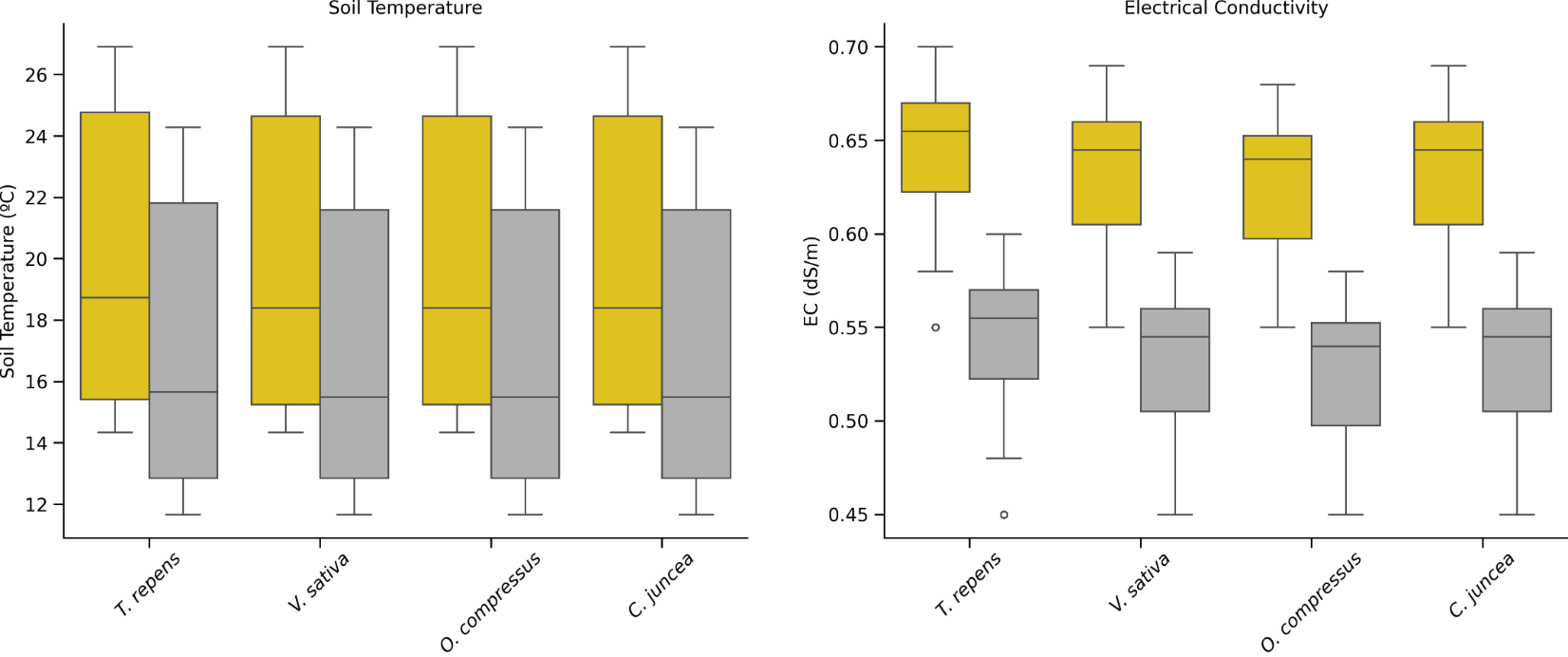


**Figure S1**. Soil temperature (ºC) and electrical conductivity (dS m⁻¹) measured across the four legume species under contrasting light treatments. Boxplots show median and interquartile ranges for each group. Grey bars correspond to plants grown in shade, whereas yellow bars represent plants grown under full light.


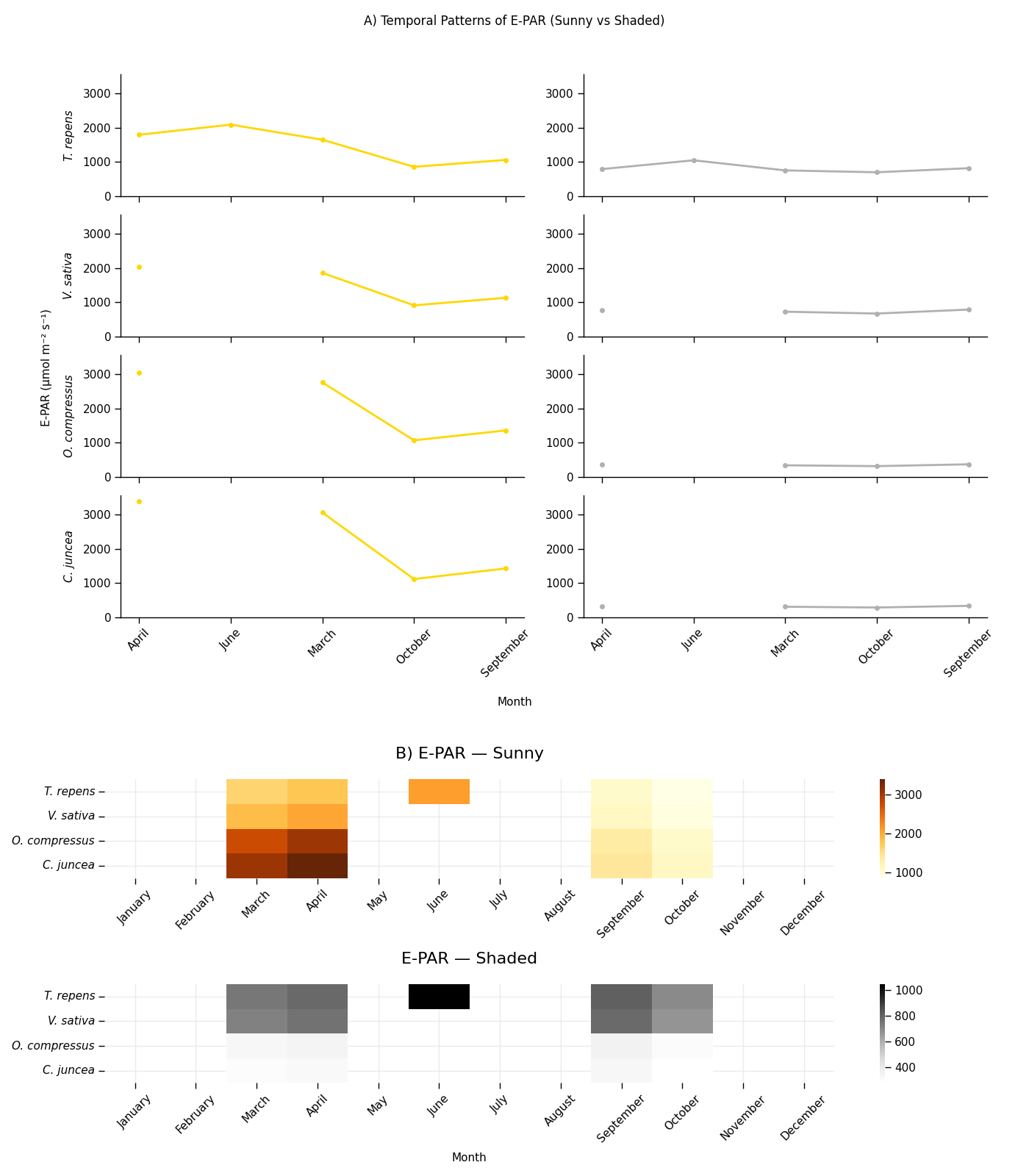


**Figure S2.** Temporal dynamics and monthly distribution of effective photosynthetically active radiation (E‑PAR) across four legume species and two light environments.

(A) Mean monthly E‑PAR for each species under sunny (yellow) and shaded (grey) treatments, shown as temporal profiles. (B) Heatmaps of mean E‑PAR unders (top) and shaded (bottom) treatments, with species arranged from top to bottom as *T. repens*, *V. sativa*, *O. compressus*, and *C. juncea*.
